# Halogen Bond Motifs in Cocrystals of *N*,*N*,*O* and *N*,*O*,*O* Acceptors Derived from Diketones and Containing a Morpholine or Piperazine Moiety

**DOI:** 10.1021/acs.cgd.2c00665

**Published:** 2022-08-01

**Authors:** Ruđer Sušanj, Vinko Nemec, Nikola Bedeković, Dominik Cinčić

**Affiliations:** Department of Chemistry, Faculty of Science, University of Zagreb, Horvatovac 102a, 10000 Zagreb, Croatia

## Abstract

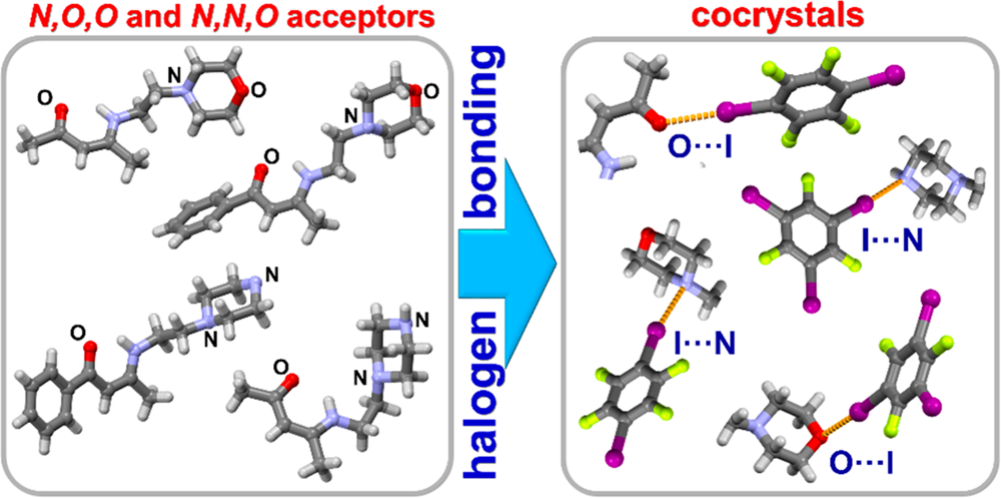

In this study, we investigate the halogen bond acceptor potential of oxygen and nitrogen atoms of morpholine and piperazine fragments when they are peripherally located on *N*,*O*,*O* or *N*,*N*,*O* acceptor molecules. We synthesized four acceptor molecules derived from either acetylacetone or benzoylacetone and cocrystallized them with 1,4-diiodotetrafluorobenzene and 1,3,5-triiodotrifluorobenzene. This resulted in eight cocrystals featuring different topicities and geometric dispositions of donor atoms. In all cocrystals, halogen bonds are formed with either the morpholinyl oxygen atom or the terminal piperazine nitrogen atom. The I···O_morpholine_ halogen bonds feature lower relative shortening values than I···N_terminal_, I···O_carbonyl_, and I···N_proximal_ halogen bonds. The N and O halogen bond acceptor sites were evaluated through calculations of molecular electrostatic potential values.

## Abstract 2

Halogen bond acceptors with peripherally located piperazine and morpholine moieties, derived from acetylacetone and benzoylacetone, have been cocrystallized with 1,4-diiodo- and 1,3,5-triiodotrifluorobenzene. Eight cocrystals were obtained, allowing a study of halogen bonding with *N*,*O*,*O* and *N*,*N*,*O* acceptor molecules, as well as a comparison to the calculated elecrostatic potentials.

In the last three decades, the halogen bond^1,2^ has become recognized as a valuable tool in crystal engineering.^3−5^ Because of its larger directionality (as compared to the hydrogen bond)^6,7^ and tunability achievable by changing the donor halogen atom in otherwise structurally equivalent donor molecules,^8−10^ the halogen bond found its way into a rising number of scientific studies on the synthesis and design of functional materials^11−15^ as well as organic synthesis,^16,17^ solution chemistry,^18,19^ pharmaceutical,^20−23^ and theoretical chemistry.^24,25^ The list of studied halogen bond donors and especially halogen bond acceptors grows continuously. Cyclic nitrogen atoms are the most studied and reliable acceptor species, and this is especially the case for pyridine nitrogen atoms,^26^ to the point that they are a valuable benchmark for donor evaluation^9,27,28^ and studies on acceptor competitiveness.^29,30^ In recent years, they have been followed by a variety of other, mostly nitrogen or oxygen atom containing species, such as methoxy,^31−33^ nitro,^34−37^ hydroxyl,^32,33,38^ and nitrile^32,39−41^ functional groups, and oxygen atoms in *N*-oxides.^42,43^ Some recent studies also showcased the promising halogen bond acceptor potential of nitrogen atoms in piperazine^10^ and nitrogen and oxygen atoms in morpholine^10,44^ as well as the carbonyl oxygen atom.^34,45−48^ So far, systematic studies of halogen bonding with these moieties were mainly limited to smaller building blocks. Searching the Cambridge Structural Database,^26^ one can find a small number of larger, relatively bulky building blocks that are halogen bonded via either the morpholine oxygen or piperazine nitrogen atoms. Most numerous are polyfunctional organic compounds,^49−64^ followed by metal–organic complexes^44,65−68^ and clathrates.^69,70^ Only a few of these studies^44,52,55,68−70^ have been systematically focused on halogen bonding. Therefore, we set out to further investigate the possibility of halogen bond formation and the possible diversity of halogen bond motifs in bulkier molecules containing peripherally located morpholine and piperazine fragments as potential building blocks in the design of novel halogen-bonded multicomponent solids.^15^

In this work, four novel Schiff bases (**BM**, **BP**, **AM**, **AP**; see Scheme 1) were synthesized by a condensation reaction from two diketones, benzoylacetone (**B**) and acetylacetone (**A**), and two primary amines, *N*-(2-aminoethyl)morpholine (**M**) and *N*-(2-aminoethyl)piperazine (**P**). Single crystals and solid bulk of pure acceptors were obtained only in the case of **BM**. Each of these molecules has three potential halogen bond acceptor sites: the carbonyl oxygen atom, nitrogen and oxygen atoms in the morpholine fragment, and the two nitrogen atoms in the piperazine fragment. An additional secondary amine nitrogen atom is also present in each of these acceptors; however, because of the geometric disposition of bonded atoms and its basicity (in comparison with the other sites, see above), this atom is not an expected acceptor site. As halogen bond donors, we selected two perfluorinated aromatic halogen bond (XB) donors, 1,4-diiodotetrafluorobenzene (**14tfib**) as a linear ditopic donor and 1,3,5-triiodo-2,4,6-trifluorobenzene (**135tfib**) as a potentially tritopic donor^28^ (Scheme 1).

**Scheme 1:**
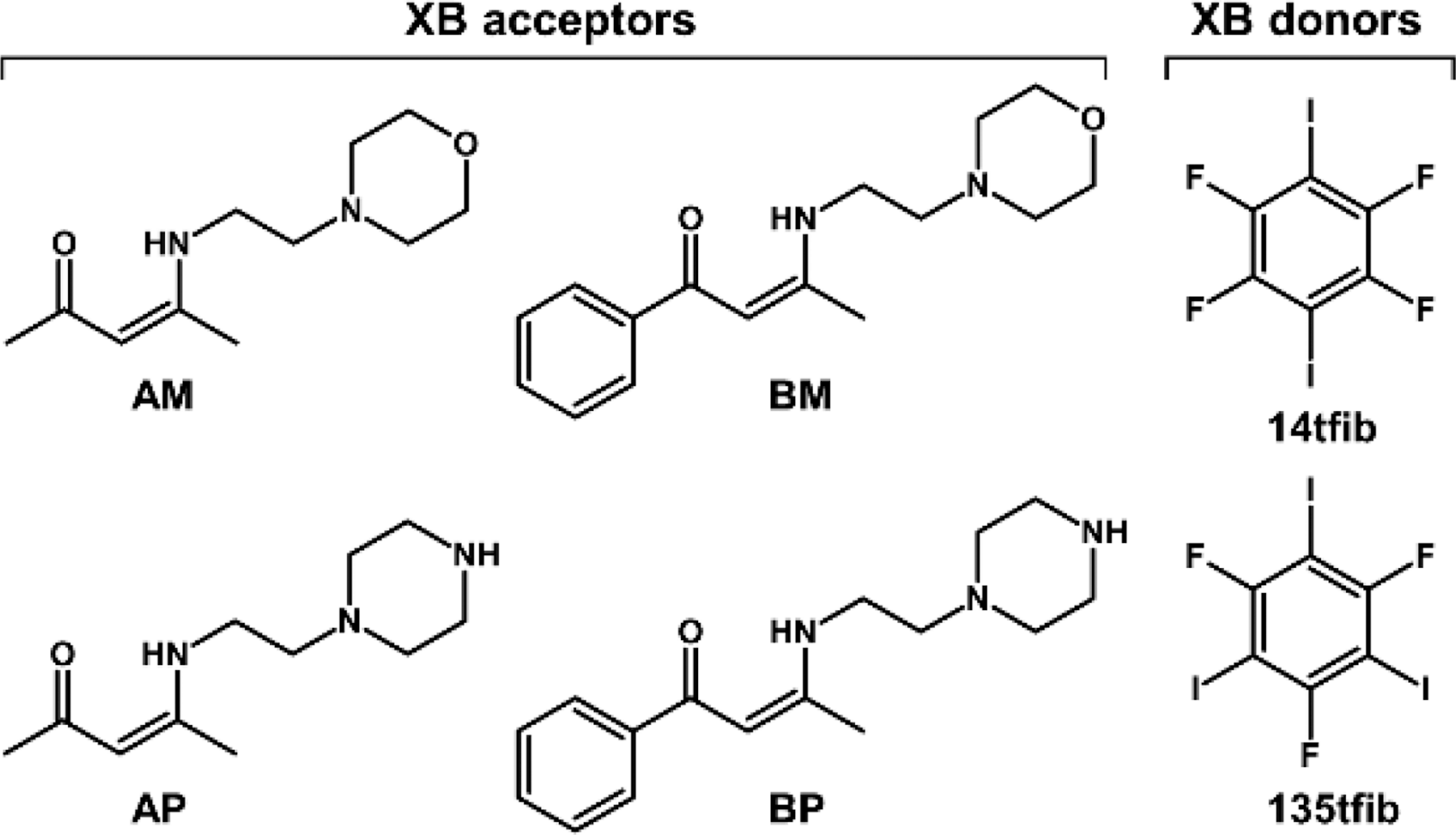
Halogen Bond Acceptor and Donor Species Used in This Study

For the purpose of ranking the acceptor sites in the acceptor molecules used in this work, the values of molecular electrostatic potentials (MEPs) were calculated on their optimized geometries (Figure 1).

**Figure 1:**
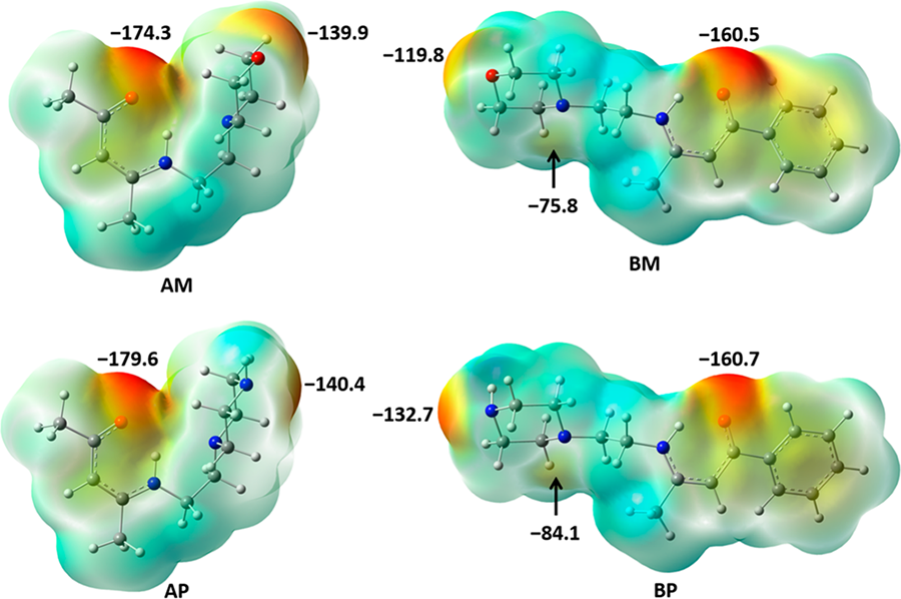
Calculated values in kJ mol^–1^*e*^–1^ of the molecular electrostatic potential mapped to the electron density isosurfaces (ρ = 0.001 a.u.) corresponding to the optimized geometries of Schiff bases **AM**, **BM**, **AP**, and **BP** (M062X/def2-tzvp level of theory).

Minima on the potential energy surfaces (PES) of the acceptors **AM** and **AP** correspond to the bent conformations, in which only two acceptor sites are available for halogen bonding. In both molecules, the MEP value on the keto oxygen atom is more negative than that on either the morpholine oxygen atom in **AM** or the piperazine nitrogen atoms in **AP**. In the case of **AP**, this difference is somewhat less pronounced, reflecting the potentially higher acceptor strength of the piperazine nitrogen atom in **AP**, compared to the morpholine oxygen atom present in **AM** (ΔMEP(**AP**) = 35 kJ mol^–1^ e^–1^, ΔMEP(**AM**) = 40 kJ mol^–1^*e*^–1^). Optimization of the extended conformation of the **AP** molecule which can be found in the (**AP**)(**135tfib**) cocrystal (see below) resulted in a geometry which corresponds to a local minimum and contains an additional acceptor site (the tertiary piperazine nitrogen atom). On the absolute scale, the MEP value of the tertiary piperazine nitrogen atom is the lowest one in the **AP** molecule and indicates the poor halogen bond acceptor strength of this moiety. After optimization, **BM** and **BP** molecules are in extended conformations, and they consequently contain three acceptor sites. On the basis of the calculated MEPs, in both molecules the best acceptor species is the keto oxygen atom, followed by either the morpholine oxygen atom (in **BM**) or the piperazine nitrogen atom (in **BP**), while the secondary amine nitrogen has been expectedly found as the weakest halogen bond acceptor site in those molecules.

Cocrystallization experiments were performed by dissolving XB donors in acceptor solutions made in an appropriate solvent or a mixture of solvents. Crystallization vessels were left at room conditions (ca. 25 °C, 40–60% RH). The obtained products were characterized by single-crystal X-ray diffraction (SCXRD), Fourier-transform infrared spectroscopy (FTIR), and thermal analysis techniques (TG–DSC). A total of eight new halogen-bonded cocrystals were obtained: (**BM**)_2_(**14tfib**)_5_, (**BM**)(**135tfib**)_2_, (**BP**)(**14tfib**), (**BP**)(**135tfib**)_2_, (**AM**)(**14tfib**), (**AM**)(**135tfib**)_2_, (**AP**)(**14tfib**), and (**AP**)(**135tfib**) (Figure 2).

**Figure 2:**
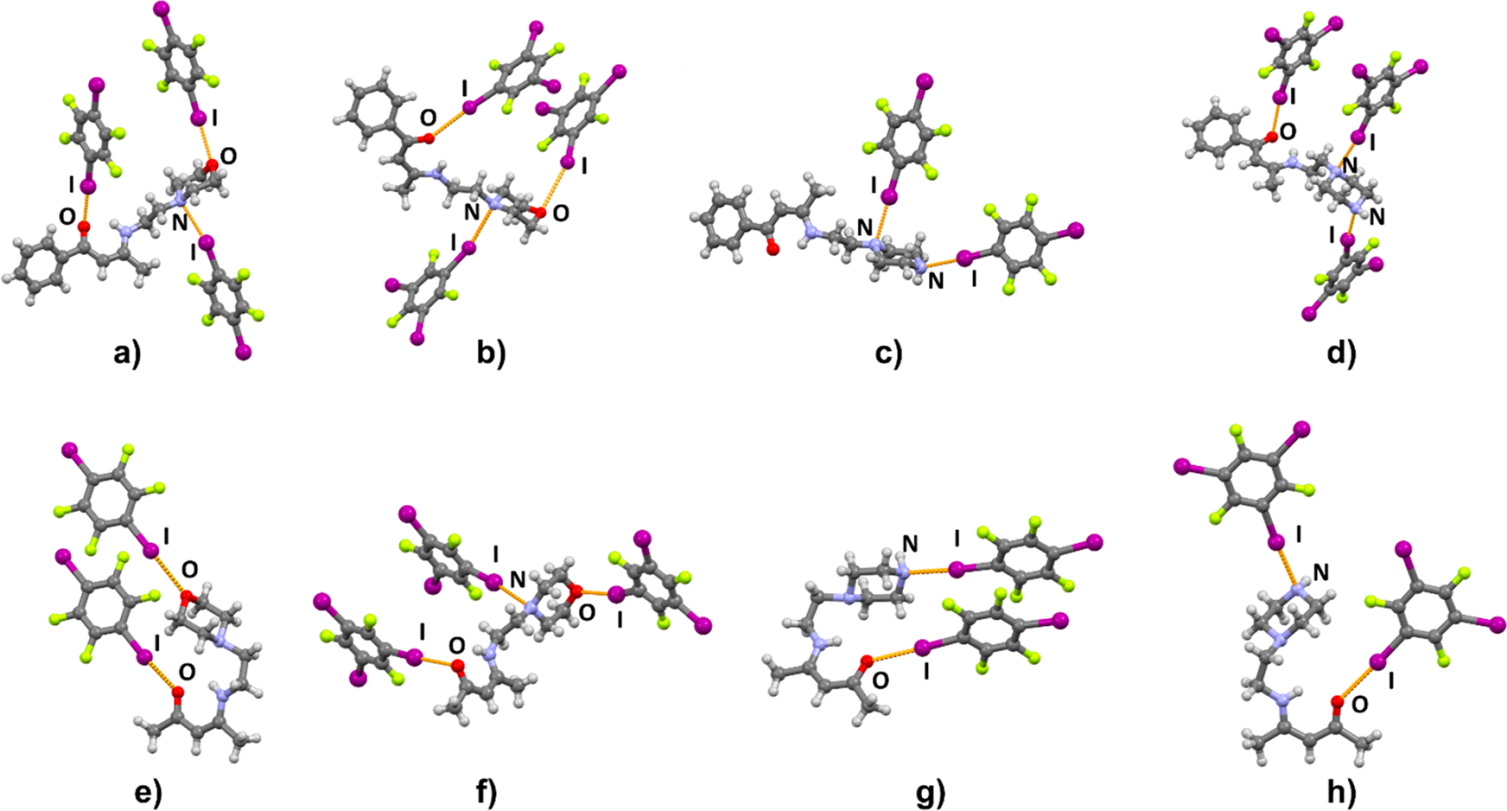
Parts of crystal structures in (a) (**BM**)_2_(**14tfib**)_5_, (b) (**BM**)(**135tfib**)_2_, (c) (**BP**)(**14tfib**), (d) (**BP**)(**135tfib**)_2_, (e) (**AM**)(**14tfib**), (f) (**AM**)(**135tfib**)_2_, (g) (**AP**)(**14tfib**), and (h) (**AP**)(**135tfib**).

As can be seen on Figure 2, halogen bonding occurs at all three targeted acceptor sites, with the additional secondary nitrogen atom not participating in it in either of the obtained cocrystals, which is in good correlation with the calculated electrostatic potentials. Of the eight cocrystals obtained, halogen bonding to the carbonyl oxygen atom (the best halogen bond acceptor site according to calculated MEPs) is present in seven (88%) of them. (**BP**)(**14tfib**) is the only cocrystal in which the carbonyl oxygen atom does not participate in halogen bonding because it is occupied in a N–H···O hydrogen bond with the piperazine nitrogen atom of an adjacent **BP** molecule. In all four morpholine-containing cocrystals, the morpholine oxygen atom is a halogen bond acceptor. Furthermore, in all four piperazine-containing cocrystals, the terminal nitrogen atom is a halogen bond acceptor, and the proximal nitrogen atoms are also acceptors in (**BP**)(**14tfib**) and (**BP**)(**135tfib**)_2_ cocrystals. In all cocrystals, the observed I···O and I···N halogen bonds exhibit interatomic distances that are at least 7% shorter than the anticipated sums of van der Waals radii^71^ (Table 1). From the crystallographic data presented in Table 1, it is noticeable that the most prominent halogen bond is the one with the terminal piperazine nitrogen atom, is present in all four obtained piperazine building block cocrystals, and features the largest relative shortening values (from 16.4% to 22.9% and averaging at 19.8%). It is followed by the I···O_carbonyl_ halogen bond, present in seven out of eight obtained cocrystals, with relative shortening values ranging from 14.4% to 20.0% and averaging at 17.5%. These *R.S.* values indicate that halogen bonds of this type can be classified as fairly strong. It can be observed that the carbonyl oxygen atom in all crystal structures forms slightly longer halogen bonds than the piperazine nitrogen atom, even though calculated MEP values on the carbonyl oxygen atoms are for the most part more negative than those on piperazine nitrogen atoms. These observed “deviations” in relative shortening values can be explained by lower steric constraints and consequently greater spatial availability of the terminal nitrogen atom relative to the carbonyl oxygen. This is especially the case in cocrystals where the acceptor molecule is in a bent conformation. Contrary to expectations based on our previous work,^44^ the I···O_morpholine_ halogen bond, although present in all four morpholine building block cocrystals, has the lowest relative shortening values, ranging between 7.3% and 15.7% and averaging at 12.9%. Somewhat stronger I···N_morpholine_ halogen bonds are present in three out of four morpholine building block cocrystals, with an average *R.S.* of 14.0%. The (**AM**)(**14tfib**) cocrystal is the only one that does not contain an I···N_morpholine_ halogen bond.

**Table 1:** Halogen Bond Lengths (*d*), Angles (∠), and Relative Shortenings (*R.S.*) of D···A Distances in the Herein Prepared Cocrystals

cocrystal	D···A	acceptor moiety	*d*(D···A)/Å	*R.S.*^a^/%	∠(C–D···A)/°
**(BM)_2_(14tfib)_5_**	I1···O1	carbonyl	2.879	17.7	177.3
	I2···O2	morpholine	2.950	15.7	179.8
	I3···N2	morpholine	3.035	14.0	169.7
**(BM)(135tfib)_2_**	I1···O1	carbonyl	2.997	14.4	175.3
	I4···N2	morpholine	3.008	14.8	165.9
	I5···O2	morpholine	3.243	7.3	143.3
**(BP)(14tfib)**	I2···N2	proximal piperazine	2.948	16.5	176.5
	I1···N3	terminal piperazine	2.760	21.8	177.3
**(BP)(135tfib)_2_**	I10···O1	carbonyl	2.917	16.7	173.6
	I7···N2	proximal piperazine	2.984	15.5	171.6
	I4···N3	terminal piperazine	2.948	16.5	171.1
	I2···O2	carbonyl	2.931	16.3	171.5
	I5···N5	proximal piperazine	2.961	16.1	173.5
	I8···N6	terminal piperazine	2.953	16.4	167.0
**(AM)(14tfib)**	I1···O1	carbonyl	2.800	20.0	174.1
	I2···O2	morpholine	3.015	13.9	177.5
**(AM)(135tfib)_2_**	I2···O1	carbonyl	2.972	15.1	173.1
	I4···O2	morpholine	3.062	12.5	168.6
	I5···N2	morpholine	3.119	11.6	173.4
	I10···O3	carbonyl	2.846	18.7	174.8
	I9···O4	morpholine	2.979	14.9	165.7
	I7···N4	morpholine	2.981	15.6	173.6
**(AP)(14tfib)**	I1···N3	terminal piperazine	2.721	22.9	176.8
	I2···O1	carbonyl	2.810	19.7	178.8
**(AP)(135tfib)**	I1···N3	terminal piperazine	2.779	21.3	176.4
	I3···O1	carbonyl	2.846	18.7	178.2
	I1···I2	135tfib	3.959	0.03	163.5

a *R.S.* = 1 – *d*(D···A)/[*r*_vdW_(D) + *r*_vdW_(A)].^71^

In all cocrystals in which it is present, **14tfib** acts as a linear ditopic donor, while **135tfib**, as expected due to its three donor atoms placed at an angle, forms structurally more intricate and diverse halogen bond motifs. Discrete halogen-bonded complexes are formed in three cocrystals with the sterically more flexible **AM** and **AP** molecules, (**AM**)(**14tfib**), (**AP**)(**14tfib**), and (**AP**)(**135tfib**), since the acceptor molecules can bend and adjust the position of their acceptor sites (Figure 3). In these cocrystals, the acceptor and donor molecules are interconnected by a combination of I···O_carbonyl_ and I···O_morpholine_ or I···N_piperazine_ halogen bonds, respectively. In the (**AM**)(**14tfib**) and (**AP**)(**14tfib**) cocrystals, the discrete complexes are connected into 3D by van der Waals contacts, while in (**AP**)(**135tfib**) they are further connected into chains via C–H···F and I···I contacts (Figure 4).

**Figure 3:**
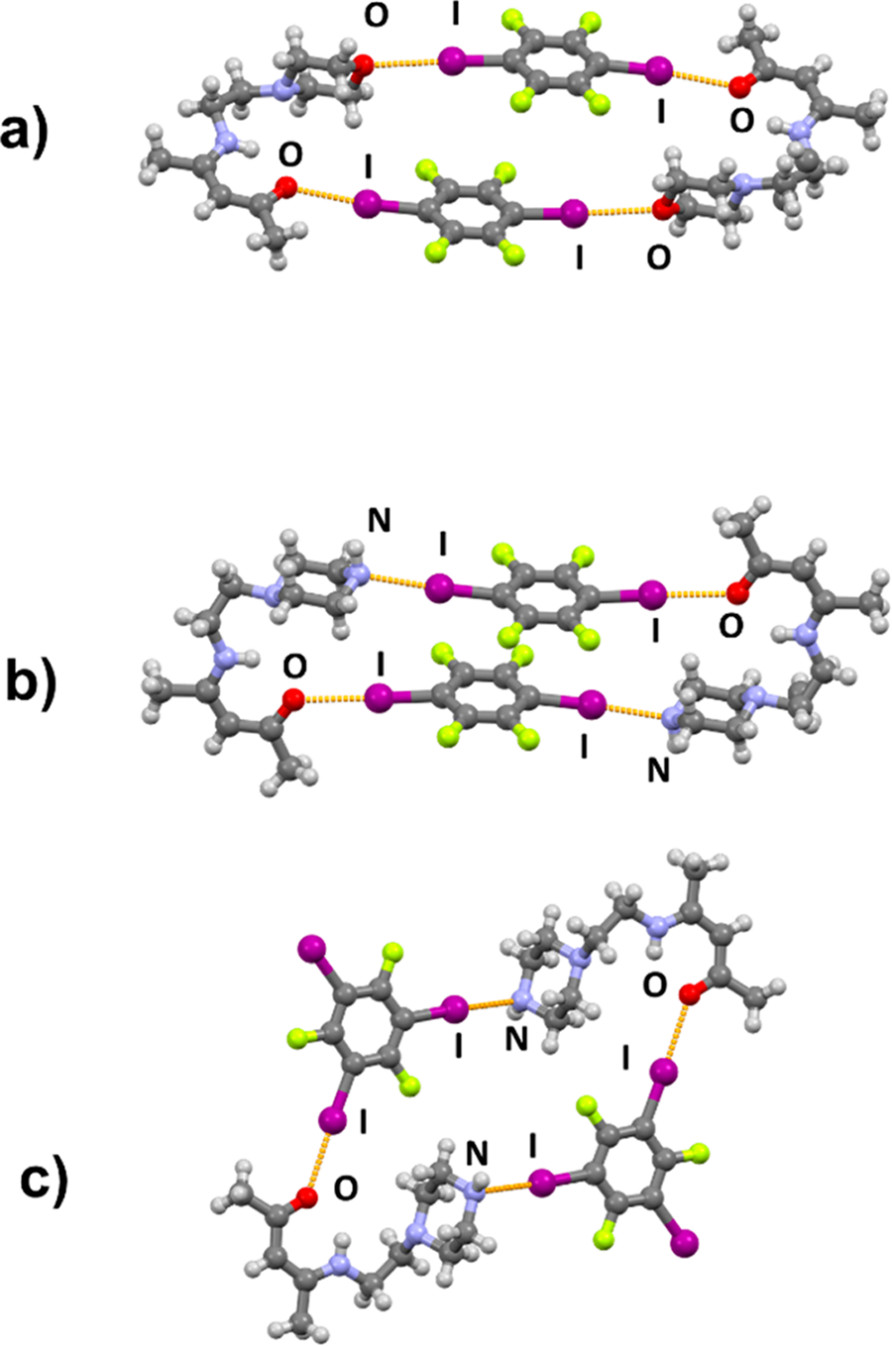
Discrete halogen-bonded complexes in crystal structures of (a) (**AM**)(**14tfib**), (b) (**AP**)(**14tfib**), and (c) (**AP**)(**135tfib**).

**Figure 4:**
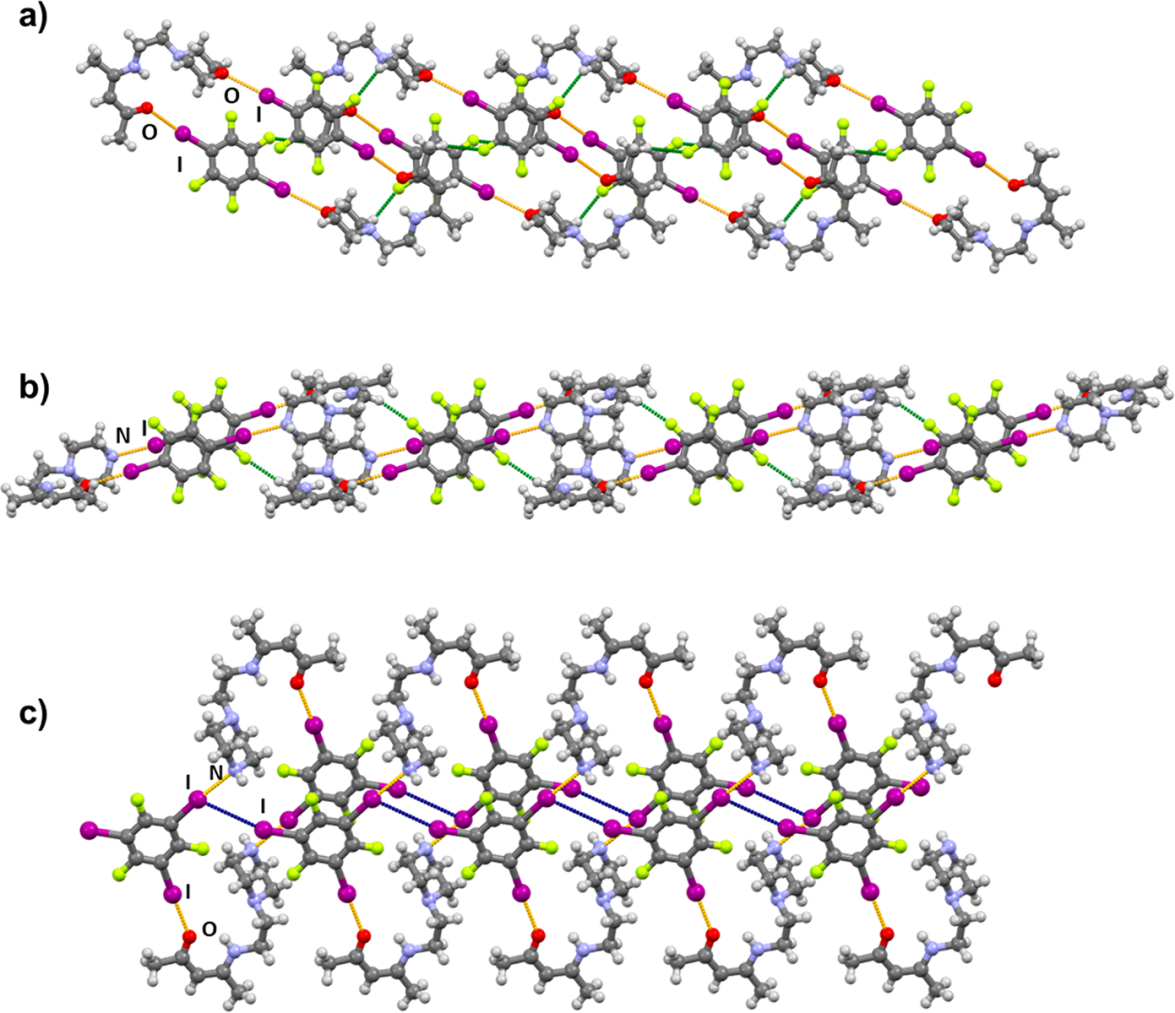
Fragments of halogen- and hydrogen-bonded chains in crystal structures of (a) (**AM**)(**14tfib**), (b) (**AP**)(**14tfib**), and (c) (**AP**)(**135tfib**). Halogen bonds (I···O and I···N) are orange, hydrogen bonds are green, and I···I halogen bonds are blue.

In cocrystals with the more sterically hindered **BM** and **BP** molecules, acceptors are connected by one or more symmetrically inequivalent halogen bond donor molecules into halogen-bonded chains. In (**BM**)_2_(**14tfib**)_5_, pairs of acceptor molecules are bridged by three **14tfib** molecules (Figure 5). A symmetrically inequivalent, nonbridging **14tfib** molecule participates in a weak I···π(C=C) halogen bond. These chains are further connected into layers by with weak I···π(C=C) halogen bonds and C–H···O_carbonyl_ and C–H··· π(phenyl) hydrogen bonds. The layers are expanded in 3D via weak C–H···F contacts.

**Figure 5:**
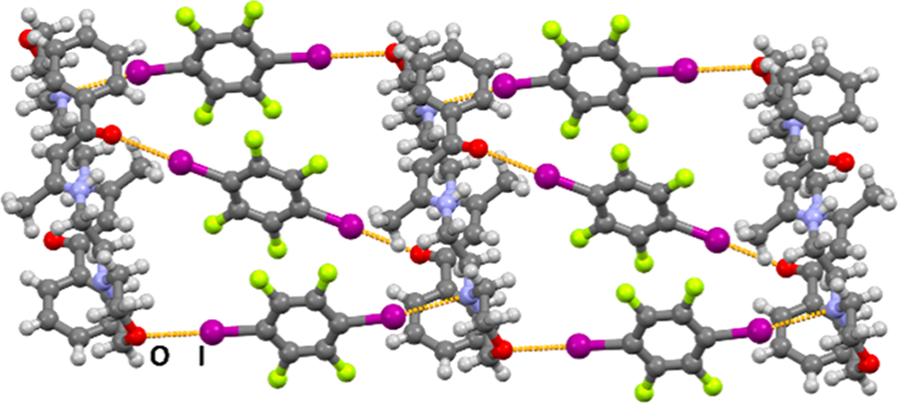
Halogen-bonded chains of (**BM**)_2_(**14tfib**)_5_.

In (**BM**)(**135tfib**)_2_, acceptor molecules are bridged by two symmetrically inequivalent **135tfib** molecules: one is halogen bonded to a morpholine oxygen atom and a morpholine nitrogen atom on an adjacent acceptor molecule, while the other **135tfib** molecule is halogen bonded to the carboxyl oxygen atom and participates in weak I···π(C=C) interactions with another acceptor molecule. The same halogen-bonding motifs can be observed in (**BP**)(**135tfib**)_2_ and (**AM**)(**135tfib**)_2_ cocrystals (Figure 6).

**Figure 6:**
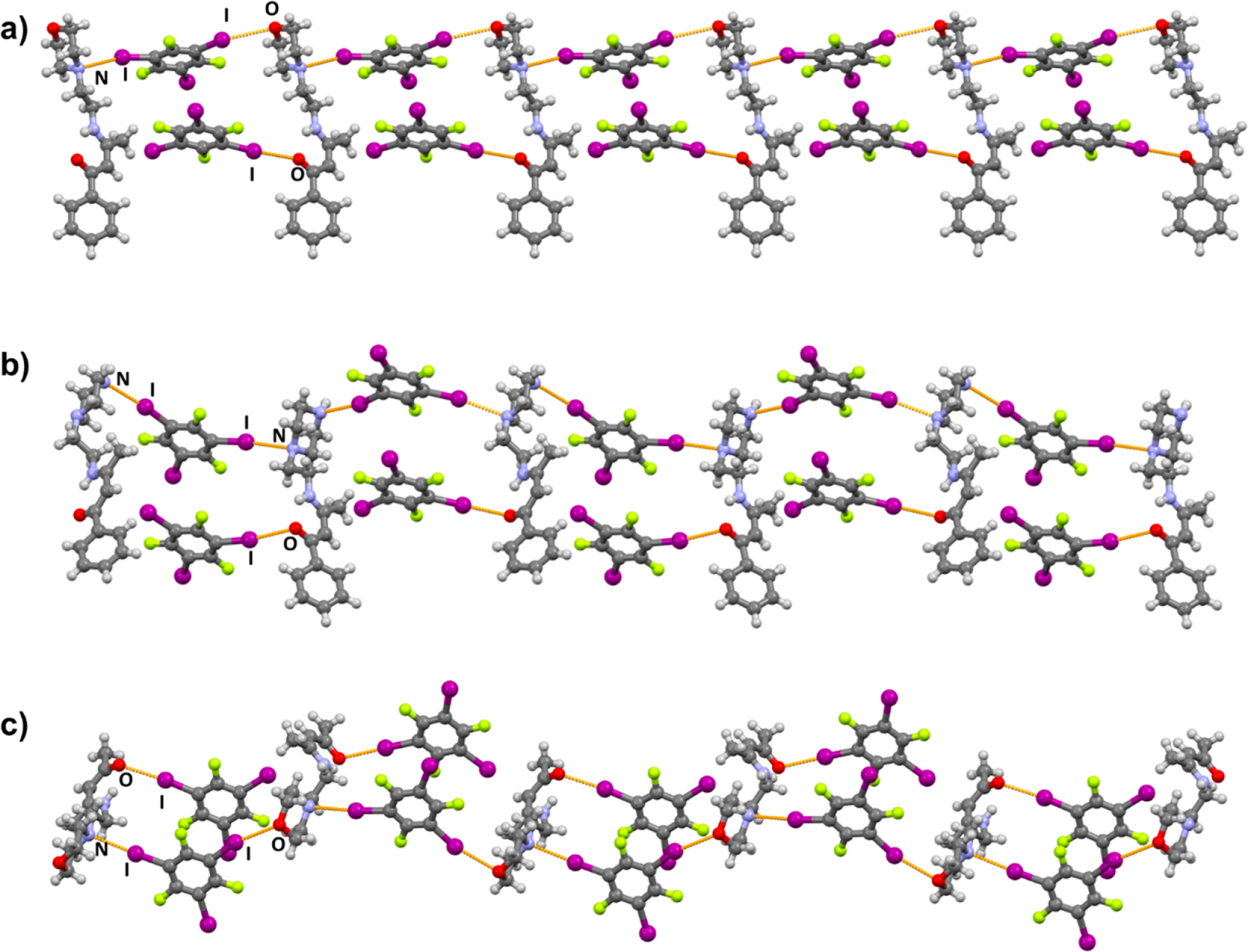
Halogen-bonded chains in crystal structures of (a) (**BM**)(**135tfib**)_2_, (b) (**BP**)(**135tfib**)_2_, and (c) (**AM**)(**135tfib**)_2_, featuring similar halogen bond motifs.

In (**BP**)(**14tfib**), the only herein presented cocrystal that does not contain halogen-bonded oxygen atoms, zigzag halogen-bonded chains are formed, in which **14tfib** molecules alternate in bridging proximal and terminal piperazine nitrogen atoms (Figure 7). The resulting chains are connected into layers through N–H···O hydrogen bonds, with the layers then connected into 3D by weak C–H···F interactions.

**Figure 7:**
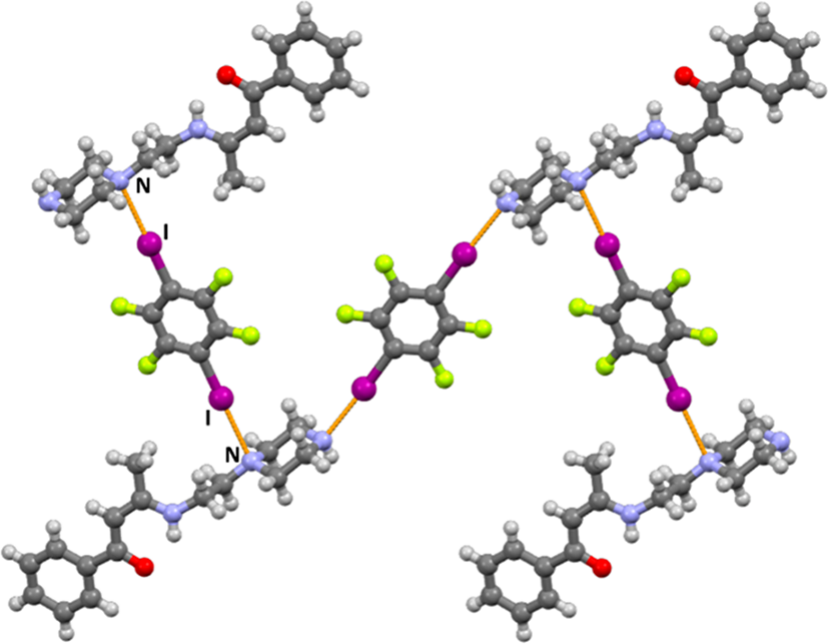
A halogen-bonded chain in the crystal structure of (**BP**)(**14tfib**).

Melting points of all of the obtained cocrystals were determined using TG-DSC analysis. For the purposes of thermal analysis, crystal bulks were synthesized by dissolving the reactants in the ratio obtained from single-crystal data. The measured PXRD patterns for the crystal bulks were found to be in good agreement with the patterns calculated from single-crystal data. Thermal analysis results are shown in Table S2 (see Supporting Information). With an exception in the (**BP**)(**14tfib**) cocrystal, which decomposes, all other cocrystals feature melting points as evidenced by a comparison of TG and DSC curves. Contrary to expectations, the melting point of the (**BM**)_2_(**14tfib**)_5_ cocrystal is the lowest of all, even though **BM** is the only acceptor obtained in the solid state. Otherwise, **BM** and **BP** cocrystals feature higher melting and decomposition point temperatures than **AM** and **AP** cocrystals.

To conclude, following our previous course of research, we have confirmed the potential of morpholine and piperazine nitrogen and oxygen atoms, as well as the carbonyl oxygen atom, as halogen bond acceptors in larger molecular building blocks by synthesizing eight cocrystals. Both the morpholinyl oxygen atom and terminal piperazine nitrogen atom were found to act as halogen bond acceptor sites in all herein presented cocrystals. However, from our analysis of crystallographic data and the comparison between relative shortening values, it is possible to infer that the terminal piperazine nitrogen atom is a better halogen bond acceptor site than the morpholinyl oxygen atom. These results could open up interesting pathways in the further development of strategies for designing novel halogen-bonded multicomponent solids, though additional studies of these bonding motifs should be performed to ascertain their reliability.

## Supporting Information Available

Experimental and computational details, including TG-DSC curves, PXRD patterns and single-crystal diffraction data (PDF)

## References

[ref1] Desiraju G. R.; Ho P. S.; Kloo L.; Legon A. C.; Marquardt R.; Metrangolo P.; Politzer P.; Resnati G.; Rissanen K. Definition of the halogen bond (IUPAC Recommendations 2013). Pure Appl. Chem. 2013, 85, 1711–1713. 10.1351/PAC-REC-12-05-10.

[ref2] Cavallo G.; Metrangolo P.; Milani R.; Pilati T.; Priimagi A.; Resnati G.; Terraneo G. The Halogen Bond. Chem. Rev. 2016, 116, 2478–2601. 10.1021/acs.chemrev.5b00484. 26812185 PMC4768247

[ref3] Mukherjee A.; Tothadi S.; Desiraju G. R. Halogen Bonds in Crystal Engineering: Like Hydrogen Bonds yet Different. Acc. Chem. Res. 2014, 47, 2514–2524. 10.1021/ar5001555. 25134974

[ref4] Li B.; Zang S.-Q.; Wang L.-Y.; Mak T. C. W. Halogen bonding: A powerful, emerging tool for constructing high-dimensional metal-containing supramolecular networks. Coord. Chem. Rev. 2016, 308, 1–21. 10.1016/j.ccr.2015.09.005.

[ref5] Bertani R.; Sgarbossa P.; Venzo A.; Lelj F.; Amati M.; Resnati G.; Pilati T.; Metrangolo P.; Terraneo G. Halogen bonding in metal-organic-supramolecular networks. Coord. Chem. Rev. 2010, 254, 677–695. 10.1016/j.ccr.2009.09.035.

[ref6] Legon A. C. The Halogen Bond: An Interim Perspective. Phys. Chem. Chem. Phys. 2010, 12, 7736–7747. 10.1039/c002129f. 20495729

[ref7] Politzer P.; Murray J. S.; Clark T. Halogen Bonding: An Electrostatically-Driven Highly Directional Noncovalent Interaction. Phys. Chem. Chem. Phys. 2010, 12, 7748–7757. 10.1039/c004189k. 20571692

[ref8] Kolář M.; Hostaš J.; Hobza P. The Strength and Directionality of a Halogen Bond Are Co-Determined by the Magnitude and Size of the σ-Hole. Phys. Chem. Chem. Phys. 2014, 16, 9987–9996. 10.1039/C3CP55188A. 24477636

[ref9] Stilinović V.; Horvat G.; Hrenar T.; Nemec V.; Cinčić D. Halogen and Hydrogen Bonding between (N-Halogeno)-succinimides and Pyridine Derivatives in Solution, the Solid State and In Silico. Chem. - Eur. J. 2017, 23, 5244–5257. 10.1002/chem.201605686. 28111817

[ref10] Cinčić D.; Friščić T.; Jones W. Isostructural Materials Achieved by Using Structurally Equivalent Donors and Acceptors in Halogen-Bonded Cocrystals. Chem.—Eur. J. 2008, 14, 747–753. 10.1002/chem.200701184. 17955560

[ref11] Dumele O.; Schreib B.; Warzok U.; Trapp N.; Schalley C. A.; Diederich F. Halogen-Bonded Supramolecular Capsules in the Solid State, in Solution, and in the Gas Phase. Angew. Chem., Int. Ed. 2017, 56, 1152–1157. 10.1002/anie.201610884. 28000334

[ref12] Borchers T. H.; Topić F.; Christopherson J.-C.; Bushuyev O. S.; Vainauskas J.; Titi H. M.; Friščić T.; Barrett C. J. Cold photo-carving of halogen-bonded co-crystals of a dye and a volatile co-former using visible light. Nat. Chem. 2022, 14, 574–581. 10.1038/s41557-022-00909-0. 35361911

[ref13] Bushuyev O. S.; Friščić T.; Barrett C. J. Controlling Dichroism of Molecular Crystals by Cocrystallization. Cryst. Growth Des. 2016, 16, 541–545. 10.1021/acs.cgd.5b01361.

[ref14] Sgarbossa P.; Bertani R.; Di Noto V.; Piga M.; Giffin G. A.; Terraneo G.; Pilati T.; Metrangolo P.; Resnati G. Interplay between Structural and Dielectric Features of New Low k Hybrid Organic–Organometallic Supramolecular Ribbons. Cryst. Growth Des. 2012, 12, 297–305. 10.1021/cg201073m.

[ref15] Nemec V.; Lisac K.; Bedeković N.; Fotović L.; Stilinović V.; Cinčić D. Crystal Engineering Strategies towards Halogen-Bonded Metal-Organic Multi-Component Solids: Salts, Cocrystals and Salt Cocrystals. CrystEngComm 2021, 23, 3063–3083. 10.1039/D1CE00158B.

[ref16] Bulfield D.; Huber S. M. Halogen Bonding in Organic Synthesis and Organocatalysis. Chem.—Eur. J. 2016, 22, 14434–14450. 10.1002/chem.201601844. 27465662

[ref17] Wolf J.; Huber F.; Erochok N.; Heinen F.; Guérin V.; Legault C. Y.; Kirsch S. F.; Huber S. M. Activation of a Metal-Halogen Bond by Halogen Bonding. Angew. Chemie - Int. Ed. 2020, 59, 16496–16500. 10.1002/anie.202005214. PMC7540446 32472957

[ref18] Beale T. M.; Chudzinski M. G.; Sarwar M. G.; Taylor M. S. Halogen Bonding in Solution: Thermodynamics and Applications. Chem. Soc. Rev. 2013, 42, 1667–1680. 10.1039/C2CS35213C. 22858664

[ref19] Erdélyi M. Halogen bonding in solution. Chem. Soc. Rev. 2012, 41, 3547–3557. 10.1039/c2cs15292d. 22334193

[ref20] Nemec V.; Vitasović T.; Cinčić D. Halogen-Bonded Cocrystals of Donepezil with Perfluorinated Diiodobenzenes. CrystEngComm 2020, 22, 5573–5577. 10.1039/D0CE01065K.

[ref21] Hernandes M. Z.; Cavalcanti S. M. T.; Moreira D. R. M.; de Azevedo Junior W. F.; Leite A. C. L. Halogen Atoms in the Modern Medicinal Chemistry: Hints for the Drug Design. Curr. Drug Targets 2010, 11, 303–314. 10.2174/138945010790711996. 20210755

[ref22] Baldrighi M.; Cavallo G.; Chierotti M. R.; Gobetto R.; Metrangolo P.; Pilati T.; Resnati G.; Terraneo G. Halogen Bonding and Pharmaceutical Cocrystals: The Case of a Widely Used Preservative. Mol. Pharmaceutics 2013, 10, 1760–1772. 10.1021/mp300574j. 23514087

[ref23] Mendez L.; Henriquez G.; Sirimulla S.; Narayan M. Looking Back, Looking Forward at Halogen Bonding in Drug Discovery. Molecules 2017, 22, 1397 10.3390/molecules22091397. PMC6151711 28837116

[ref24] Wang C.; Danovich D.; Mo Y.; Shaik S. On the Nature of the Halogen Bond. J. Chem. Theory Comput. 2014, 10, 3726–3737. 10.1021/ct500422t. 26588518

[ref25] Wang H.; Wang W.; Jin W. J. σ-Hole Bond vs π-Hole Bond: A Comparison Based on Halogen Bond. Chem. Rev. 2016, 116 (9), 5072–5104. 10.1021/acs.chemrev.5b00527. 26886515

[ref26] Groom C. R.; Bruno I. J.; Lightfoot M. P.; Ward S. C. The Cambridge Structural Database. Acta Crystallogr. Sect. B Struct. Sci. Cryst. Eng. Mater. 2016, 72 (2), 171–179. 10.1107/S2052520616003954. PMC4822653 27048719

[ref27] Nicolas I.; Barrière F.; Jeannin O.; Fourmigué M. Sequential Halogen Bonding with Ditopic Donors: σ-Hole Evolutions upon Halogen Bond Formation. Cryst. Growth Des. 2016, 16, 2963–2971. 10.1021/acs.cgd.6b00333.

[ref28] Bedeković N.; Piteša T.; Eraković M.; Stilinović V.; Cinčić D. Anticooperativity of multiple halogen bonds and its effect on stoichiometry of cocrystals of perfluorinated iodobenzenes. Cryst. Growth Des. 2022, 22, 2644–2653. 10.1021/acs.cgd.2c00077. PMC8991082 35401054

[ref29] Aakeröy C. B.; Wijethunga T. K.; Desper J.; Đaković M. Electrostatic Potential Differences and Halogen-Bond Selectivity. Cryst. Growth Des. 2016, 16, 2662–2670. 10.1021/acs.cgd.5b01770.

[ref30] Baus Topić N.; Bedeković N.; Lisac K.; Stilinović V.; Cinčić D. Halogen-Bonded Cocrystals of 1,3,5-Triiodo-2,4,6-trifluorobenzene and Structural Isomers of Benzoylpyridine. Cryst. Growth Des. 2022, 22, 3981–3989. 10.1021/acs.cgd.2c00382.

[ref31] Präsang C.; Whitwood A. C.; Bruce D. W. Spontaneous symmetry-breaking in halogen-bonded, bent-core liquid crystals: observation of a chemically driven Iso–N–N* phase sequence. Chem. Commun. 2008, 2137–2139. 10.1039/b719555a. 18438494

[ref32] Zbačnik M.; Vitković M.; Vulić V.; Nogalo I.; Cinčić D. Competition between Halogen Bonds in Cocrystals of Imines Derived from o-Vanillin. Cryst. Growth Des. 2016, 16, 6381–6389. 10.1021/acs.cgd.6b01037.

[ref33] Carletta A.; Spinelli F.; d’Agostino S.; Ventura B.; Chierotti M. R.; Gobetto R.; Wouters J.; Grepioni F. Halogen-Bond Effects on the Thermo- and Photochromic Behaviour of Anil-Based Molecular Co-crystals. Chem.—Eur. J. 2017, 23, 5317–5329. 10.1002/chem.201605953. 28240437

[ref34] Nemec V.; Cinčić D. Uncommon halogen bond motifs in cocrystals of aromatic amines and 1,4-diiodotetrafluorobenzene. CrystEngComm 2016, 18, 7425–7429. 10.1039/C6CE01703G.

[ref35] Aakeröy C. B.; Chopade P. D.; Desper J. Avoiding “Synthon Crossover” in Crystal Engineering with Halogen Bonds and Hydrogen Bonds. Cryst. Growth Des. 2011, 11, 5333–5336. 10.1021/cg2009013.

[ref36] Saha B. K.; Nangia A.; Jaskólski M. Crystal engineering with hydrogen bonds and halogen bonds. CrystEngComm 2005, 7, 355–358. 10.1039/b501693b.

[ref37] Tothadi S.; Sanphui P.; Desiraju G. R. Obtaining Synthon Modularity in Ternary Cocrystals with Hydrogen Bonds and Halogen Bonds. Cryst. Growth Des. 2014, 14, 5293–5302. 10.1021/cg501115k.

[ref38] Zbačnik M.; Pajski M.; Stilinović V.; Vitković M.; Cinčić D. The halogen bonding proclivity of the ortho-methoxy–hydroxy group in cocrystals of o-vanillin imines and diiodotetrafluoro-benzenes. CrystEngComm 2017, 19, 5576–5582. 10.1039/C7CE01332A.

[ref39] Aakeröy C. B.; Spartz C. L.; Dembowski S.; Dwyre S.; Desper J. A systematic structural study of halogen bonding versus hydrogen bonding within competitive supramolecular systems. IUCrJ. 2015, 2, 498–510. 10.1107/S2052252515010854. 26306192 PMC4547818

[ref40] Szell P. M. J.; Gabriel S. A.; Caron-Poulin E.; Jeannin O.; Fourmigué M.; Bryce D. L. Cosublimation: A Rapid Route Toward Otherwise Inaccessible Halogen-Bonded Architectures. Cryst. Growth Des. 2018, 18, 6227–6238. 10.1021/acs.cgd.8b01089.

[ref41] Torubaev Y. V.; Skabitsky I. V. The energy frameworks of aufbau synthon modules in 4-cyanopyridine co-crystals. CrystEngComm 2019, 21, 7057–7068. 10.1039/C9CE01174A.

[ref42] Puttreddy R.; Rautiainen J. M.; Mäkelä T.; Rissanen K. Strong N–X···O–N Halogen Bonds: A Comprehensive Study on N-Halosaccharin Pyridine N-Oxide Complexes. Angew. Chem., Int. Ed. 2019, 58, 18610–18618. 10.1002/anie.201909759. 31613414

[ref43] Aakeröy C. B.; Wijethunga T. K.; Desper J. Constructing molecular polygons using halogen bonding and bifurcated N-oxides. CrystEngComm 2014, 16, 28–31. 10.1039/C3CE41887A.

[ref44] Nemec V.; Piteša T.; Friščić T.; Cinčić D. The Morpholinyl Oxygen Atom as an Acceptor Site for Halogen-Bonded Cocrystallization of Organic and Metal-Organic Units. Cryst. Growth Des. 2020, 20, 3617–3624. 10.1021/acs.cgd.0c00520.

[ref45] Nemec V.; Fotović L.; Vitasović T.; Cinčić D. Halogen Bonding of the Aldehyde Oxygen Atom in Cocrystals of Aromatic Aldehydes and 1,4-Diiodotetrafluorobenzene. CrystEngComm 2019, 21, 3251–3255. 10.1039/C9CE00340A.

[ref46] Syssa-Magalé J.-L.; Boubekeur K.; Schöllhorn B. First molecular self-assembly of 1,4-diiodo-tetrafluoro-benzene and a ketone via (O···I) non-covalent halogen bonds. J. Mol. Struct. 2005, 737, 103–107. 10.1016/j.molstruc.2004.10.008.

[ref47] Cinčić D.; Friščić T. Synthesis of an extended halogen-bonded metal–organic structure in a one-pot mechanochemical reaction that combines covalent bonding, coordination chemistry and supramolecular synthesis. CrystEngComm 2014, 16, 10169–10172. 10.1039/C4CE01815J.

[ref48] Bedeković N.; Fotović L.; Stilinović V.; Cinčić D. Conservation of the Hydrogen-Bonded Pyridone Homosynthon in Halogen-Bonded Cocrystals. Cryst. Growth Des. 2022, 22, 987–992. 10.1021/acs.cgd.1c01424. PMC8861932 35210955

[ref49] Guo M.-L.; Zhang L. 2,4-Dibromo-6-[(4-methylpiperazin-1-yl)iminomethyl]phenol. Acta Crystallogr. Sect. E: Struct 2007, 63, o4558 10.1107/S1600536807054335.

[ref50] Jayamani A.; Thamilarasan V.; Sengottuvelan N.; Manisankar P.; Kang S. K.; Kim Y. I.; Ganesan V. Synthesis of Mononuclear Copper(II) Complexes of Acyclic Schiff’s Base Ligands: Spectral, Structural, Electrochemical, Antibacterial, DNA Binding and Cleavage Activity. Spectrochim. Acta Part A Mol. Biomol. Spectrosc. 2014, 122, 365–374. 10.1016/j.saa.2013.11.079. 24317263

[ref51] Copolovici L.; Bojan V.; Silvestru C.; Varga R. A. 1-Bromo-2,6-bis(N-morpholinylmethyl)benzene. Acta Crystallogr. Sect. E: Struct. 2007, 63, o4570 10.1107/S1600536807051574.

[ref52] Bushuyev O. S.; Tan D.; Barrett C. J.; Friščić T. Fluorinated azobenzenes with highly strained geometries for halogen bond-driven self-assembly in the solid state. CrystEngComm 2015, 17, 73–80. 10.1039/C4CE01216J.

[ref53] Modarresi-Alam A. R.; Amirazizi H. A.; Bagheri H.; Bijanzadeh H. R.; Kleinpeter E. Dynamic 1H NMR Spectroscopic Study of the Ring Inversion in N-Sulfonyl Morpholines - Studies on N-S Interactions. J. Org. Chem. 2009, 74, 4740–4746. 10.1021/jo900454a. 19558179

[ref54] Sergienko V. S.; Abramenko V. L.; Churakov A. V.; Surazhskaya M. D. Benzenoid-quinoid tautomerism of salicylidenimines: crystal structures of 5-Bromo-(HL1) and 3-nitro-salicylidene- (2-morpholino)ethylimine (HL2). J. Struct. Chem. 2021, 62, 436–442. 10.1134/S0022476621030100.

[ref55] Li C.; Chai Y.; Zhou X.; Shen Z.; Ma B.; Chen B.; Huang R.; Chen H.; Li W.; He Y. Choice of hydrogen bonds or halogen bonds by 2-halogenated 5-morpholinomethylphenyl triazolo[1,5-*a*]pyridine. CrystEngComm 2018, 20 (22), 3006–3010. 10.1039/C8CE00423D.

[ref56] Hassanain H.; Davies E. S.; Lewis W.; Kays D. L.; Champness N. R. Morpholino-Substituted BODIPY Species: Synthesis, Structure and Electrochemical Studies. Crystals 2020, 10, 36 10.3390/cryst10010036.

[ref57] Lei N.; Shen Y.; Li Y.; Tao P.; Yang L.; Su Z.; Zheng K. Electrochemical Iodoamination of Indoles Using Unactivated Amines. Org. Lett. 2020, 22, 9184–9189. 10.1021/acs.orglett.0c03158. 33185451

[ref58] Bahrin L. G.; Hrib C. G.; Birsa L. M. 4-Bromo-2-[5-methyl-2-(morpholin-4-yl)-1,3-thiazol-4-yl]phenol. Acta Crystallogr. Sect. E Struct. 2013, 69, o1170 10.1107/S1600536813017510. PMC3770429 24046714

[ref59] Sempere Y.; Alfke J. L.; Rössler S. L.; Carreira E. M. Morpholine Ketene Aminal as Amide Enolate Surrogate in Iridium-Catalyzed Asymmetric Allylic Alkylation. Angew. Chem. 2019, 131 (28), 9637–9641. 10.1002/ange.201903090. 31069899

[ref60] Ma L.; Li A. M.; Wan C. Q.; Cao S. L. Crystal Structure of (Z)-Methyl N’-(5,7-dibromo-1-(2-morpholinoethyl)-2-oxoindolin-3-ylidene)hydrazinecarbodithioate, C16H18Br2N4O2S2. Z. Kristallogr. - New Cryst. Struct. 2014, 229, 25–26. 10.1515/ncrs-2014-0020.

[ref61] Honraedt A.; Van Der Lee A.; Campagne J. M.; Leclerc E. α,α-Difluoro-α-(Trimethylsilyl)Acetamides as Versatile Reagents for the Preparation of Difluorinated Aldol and Mannich Adducts. Adv. Synth. Catal. 2017, 359, 2815–2823. 10.1002/adsc.201700371.

[ref62] Cunha S.; Oliveira C. C; Sabino J. R Synthesis of 3-Bromotetronamides via Amination of 3,4-Dibromofuran-2(5H)-One. J. Braz. Chem. Soc. 2011, 22, 598–603. 10.1590/S0103-50532011000300026.

[ref63] Haldón E.; Álvarez E.; Carmen Nicasio M.; Pérez P. J. 1,2,3-Triazoles from Carbonyl Azides and Alkynes: Filling the Gap. Chem. Commun. 2014, 50, 8978–8981. 10.1039/C4CC03614J. 24980244

[ref64] Ibis C.; Deniz N. G.; Tuyun A. F. Crystal Structure of 4-Bromo-3,4-Dichloro-1-(1-Morpholinyl)-1-(Decylsulfanyl)-2-Nitro-Buta-1,3-Diene. J. Chem. Crystallogr. 2010, 40, 353–356. 10.1007/s10870-009-9660-7.

[ref65] Davidson M. G.; Johnson A. L.; Jones M. D.; Lunn M. D.; Mahon M. F. Titanium(IV) Complexes of Hydrazones and Azines. Eur. J. Inorg. Chem. 2006, 2006, 4449–4454. 10.1002/ejic.200600501.

[ref66] Demakova M. Y.; Bolotin D. S.; Bokach N. A.; Islamova R. M.; Starova G. L.; Kukushkin V. Y. Click-Type Pt^II^-Mediated Hydroxyguanidine-Nitrile Coupling Provides Useful Catalysts for Hydrosilylation Cross-Linking. ChemPlusChem. 2015, 80, 1607–1614. 10.1002/cplu.201500327. 31973364

[ref67] Xu S. P.; Shi L.; Lv P. C.; Fang R. Q.; Zhu H. L. Synthesis and Antibacterial Activities of Metal(II) Complexes with Schiff Bases Derived from 3,5-Diiodosalicylaldehyde. J. Coord. Chem. 2009, 62, 2048–2057. 10.1080/00958970902741251.

[ref68] Lapadula G.; Judaš N.; Friščić T.; Jones W. A Three-Component Modular Strategy to Extend and Link Coordination Complexes by Using Halogen Bonds to O, S and π Acceptors. Chem. - A Eur. J. 2010, 16, 7400–7403. 10.1002/chem.201000049. 20486234

[ref69] Raatikainen K.; Huuskonen J.; Lahtinen M.; Metrangolo P.; Rissanen K. Halogen Bonding Drives the Self-Assembly of Piperazine Cyclophanes into Tubular Structures. Chem. Commun. 2009, 2160–2162. 10.1039/b901473j. 19360179

[ref70] Raatikainen K.; Rissanen K. Modulation of N···I and ^+^N−H···Cl^−^···I Halogen Bonding: Folding, Inclusion, and Self-Assembly of Tri-and Tetraamino Piperazine Cyclophanes. Cryst. Growth Des. 2010, 10, 3638–3646. 10.1021/cg100516t.

[ref71] Bondi A. Van Der Waals Volumes and Radii. J. Phys. Chem. 1964, 68, 441–451. 10.1021/j100785a001.

