# Supplementary material for: Halogen Bond Motifs in Cocrystals of N,N,O and N,O,O Acceptors Derived from Diketones and Containing a Morpholine or Piperazine Moiety
Source: Cryst Growth Des. 2022 Aug 1;22(9):5135–42. doi: 10.1021/acs.cgd.2c00665 (PMC9461725; doi:10.1021/acs.cgd.2c00665)
Supplement: Supplementary file 1 — cg2c00665_si_001.pdf [file cg2c00665_si_001.pdf]

SUPPORTING INFORMATION

Halogen bond motifs in cocrystals of *N,N,O* and *N,O,O* acceptors derived from diketones and containing a morpholine or piperazine moiety

*Ruđer Sušanj,[1] Vinko Nemec, [1] Nikola Bedeković,[1] and Dominik Cinčić[1]**

[1] Department of Chemistry, Faculty of Science, University of Zagreb, Horvatovac 102a, HR-10000 Zagreb, Croatia

Email: dominik@chem.pmf.hr
Fax: +385 1 4606 341
Tel: +385 1 4606 362

Table of Contents

Experimental details	Preparation of imines, solution syntheses of cocrystals and crystal bulks, thermal analysis, FTIR spectroscopy, powder and single crystal X-ray diffraction experiments, computational details	3
Table S1.	Crystal data and refinement details for the prepared cocrystals.	8
Table S2.	Melting point and decomposition signal onset temperatures, t_E	13
Figure S1.	Partial molecular structure of **BM** showing the atom-labelling scheme. Displacement ellipsoids are drawn at the 50 % probability level, and H atoms are shown as small spheres of arbitrary radius.	14
Figure S2.	Partial molecular structure of (**BM**)$_2$(**14tfib**)$_5$ showing the atom-labelling scheme. Displacement ellipsoids are drawn at the 50 % probability level, and H atoms are shown as small spheres of arbitrary radius.	14
Figure S3.	Partial molecular structure of (**BM**)(**135tfib**)$_2$ showing the atom-labelling scheme. Displacement ellipsoids are drawn at the 50 % probability level, and H atoms are shown as small spheres of arbitrary radius.	15
Figure S4.	Partial molecular structure of (**BP**)(**14tfib**) showing the atom-labelling scheme. Displacement ellipsoids are drawn at the 50 % probability level, and H atoms are shown as small spheres of arbitrary radius.	15
Figure S5.	Partial molecular structure of (**BP**)(**135tfib**)$_2$ showing the atom-labelling scheme. Displacement ellipsoids are drawn at the 50 % probability level, and H atoms are shown as small spheres of arbitrary radius.	16
Figure S6.	Partial molecular structure of (**AM**)(**14tfib**) showing the atom-labelling scheme. Displacement ellipsoids are drawn at the 50 % probability level, and H atoms are shown as small spheres of arbitrary radius.	16
Figure S7.	Partial molecular structure of (**AM**)(**135tfib**)$_2$ showing the atom-labelling scheme. Displacement ellipsoids are drawn at the 50 % probability level, and H atoms are shown as small spheres of arbitrary radius.	17
Figure S8.	Partial molecular structure of (**AP**)(**14tfib**) showing the atom-labelling scheme. Displacement ellipsoids are drawn at the 50 % probability level, and H atoms are shown as small spheres of arbitrary radius.	17
Figure S9.	Partial molecular structure of (**AP**)(**135tfib**) showing the atom-labelling scheme. Displacement ellipsoids are drawn at the 50 % probability level, and H atoms are shown as small spheres of arbitrary radius.	18
Figure S10.	PXRD patterns of **BM**.	19
Figure S11.	PXRD patterns of (**BM**)$_2$(**14tfib**)$_5$.	19

Figure S12. PXRD patterns of (**BM**)(**135tfib**)$_2$ 20
Figure S13. PXRD patterns of (**BP**)(**14tfib)**. 20
Figure S14. PXRD patterns of (**BP**)(**135tfib**)$_2$. 21
Figure S15. PXRD patterns of (**AM**)(**14tfib**). 21
Figure S16. PXRD patterns of (**AM**)(**135tfib**)$_2$. 22
Figure S17. PXRD patterns of (**AP**)(**14tfib**). 22
Figure S18. PXRD patterns of (**AP**)(**135tfib**). 23
Figure S19. TG-DSC curve of **BM**. 24
Figure S20. TG-DSC curve of (**BM**)$_2$(**14tfib**)$_5$. 24
Figure S21. TG-DSC curve of (**BM**)(**135tfib**)$_2$ 25
Figure S22. TG-DSC curve of (**BP**)(**14tfib**). 25
Figure S23. TG-DSC curve of (**BP**)(**135tfib**)$_2$. 26
Figure S24. TG-DSC curve of (**AM**)(**14tfib**). 26
Figure S25. TG-DSC curve of (**AM**)(**135tfib**)$_2$. 27
Figure S26. TG-DSC curve of (**AP**)(**14tfib**). 27
Figure S27. TG-DSC curve of (**AP**)(**135tfib**). 28
Figure S28. IR spectrum of **BM** from 110 to 800 cm^{-1}. 29
Figure S29. IR spectrum of **14tfib** from 110 to 800 cm^{-1}. 29
Figure S30. IR spectrum of **135tfib** from 110 to 800 cm^{-1}. 29
Figure S31. IR spectrum of (**BM**)$_2$(**14tfib**)$_5$ from 110 to 800 cm^{-1}. 30
Figure S32. IR spectrum of (**BM**)(**135tfib**)$_2$ from 110 to 800 cm^{-1}. 30
Figure S33. IR spectrum of (**BP**)(**14tfib**) from 110 to 800 cm^{-1}. 30
Figure S34. IR spectrum of (**BP**)(**135tfib**)$_2$ from 110 to 800 cm^{-1}. 31
Figure S35. IR spectrum of (**AM**)(**14tfib**) from 110 to 800 cm^{-1}. 31
Figure S36. IR spectrum of (**AM**)(**135tfib**)$_2$ from 110 to 800 cm^{-1}. 31
Figure S37. IR spectrum of (**AP**)(**14tfib**) from 110 to 800 cm^{-1}. 32
Figure S38. IR spectrum of (**AP**)(**135tfib**) from 110 to 800 cm^{-1}. 32
Figure S39. Optimized geometry of **AM** (bent). 32
Figure S40. Optimized geometry of **AM** (extended conformation). 33
Figure S41. Optimized geometry of **AP**. 33
Figure S42. Optimized geometry of **BM**. 33
Figure S43. Optimized geometry of **BP**. 33

EXPERIMENTAL DETAILS

Preparation of **BM**

811 mg (5.00 mmol) of benzoylacetone was dissolved in 5.0 mL of methanol, followed by the addition of 655 μL (5.00 mmol) of 1-(2-aminoethyl)morpholine. The solution was left to crystallize at room temperature for a day.

Preparation of **BP**

811 mg (5.00 mmol) of benzoylacetone was dissolved in 5.0 mL of methanol, followed by the addition of 655 μL (5.00 mmol) of 1-(2-aminoethyl)piperazine. This solution of 1.00 mmol mL^{-1} was then used in crystallization experiments.

Preparation of **AM**

510 μL (5.00 mmol) of acetylacetone was dissolved in 5.0 mL of methanol, followed by the addition of 655 μL (5.00 mmol) of 1-(2-aminoethyl)morpholine. This solution of 1.00 mmol mL^{-1} was then used in crystallization experiments.

Preparation of **AP**

510 μL (5.00 mmol) of acetylacetone was dissolved in 5.0 mL of methanol, followed by the addition of 655 μL (5.00 mmol) of 1-(2-aminoethyl)piperazine. This solution of 1.00 mmol mL^{-1} was then used in crystallization experiments.

SOLUTION SYNTHESES

Single crystals of **(BM)**$_2$**(14tfib)**$_5$

A mixture of **BM** (27.4 mg, 0.100 mmol) and **14tfib** (40.1 mg, 0.100 mmol) was dissolved in a solvent mixture of 2.0 mL diethyl ether and 2.0 mL petroleum ether and left to crystallize at room temperature.

Single crystals of **(BM)(135tfib)**$_2$

A mixture of **BM** (27.4 mg, 0.100 mmol) and **135tfib** (51.0 mg, 0.100 mmol) was dissolved in a solvent mixture of 1.0 mL methanol and 1.0 mL acetonitrile and left to crystallize at room temperature.

Single crystals of **(BP)(14tfib)**

A 100 μL aliquot of the **BP** solution (0.100 mmol) was diluted in a solvent mixture of 1.0 mL methanol and 1.0 mL acetonitrile, followed by the dissolution of 40.1 mg of **14tfib** (0.100 mmol). The solution was then left to crystallize at room temperature.

Single crystals of **(BP)(135tfib)**$_2$

A 100 μL aliquot of the **BP** solution (0.100 mmol) was diluted in a solvent mixture of 1.0 mL methanol and 1.0 mL acetonitrile, followed by the dissolution of 51.0 mg of **135tfib** (0.100 mmol). The solution was then left to crystallize at room temperature.

Single crystals of **(AM)(14tfib)**

A 100 μL aliquot of the **AM** solution (0.100 mmol) was diluted in 2 mL of tetrahydrofuran, followed by the dissolution of 40.1 mg of **14tfib** (0.100 mmol). The solution was then left to crystallize at room temperature.

Single crystals of **(AM)(135tfib)**$_2$

A 100 μL aliquot of the **AM** solution (0.100 mmol) was diluted in 2 mL of tetrahydrofuran, followed by the dissolution of 51.0 mg of **135tfib** (0.100 mmol). The solution was then left to crystallize at room temperature.

Single crystals of **(AP)(14tfib)**

A 100 μL aliquot of the **AP** solution (0.100 mmol) was diluted in a solvent mixture of 1.0 mL methanol and 1.0 mL acetonitrile, followed by the dissolution of 40.1 mg of **14tfib** (0.100 mmol). The solution was then left to crystallize at room temperature.

Single crystals of **(AP)(135tfib)**

A 100 μL aliquot of the **AP** solution (0.100 mmol) was diluted in 2 mL of tetrahydrofuran, followed by the dissolution of 51.0 mg of **135tfib** (0.100 mmol). The solution was then left to crystallize at room temperature.

Solution syntheses of crystal bulk products used for thermal analysis and FTIR spectroscopy

Crystal bulk of **(BM)(14tfib)**

A mixture of **BM** (27.4 mg, 0.100 mmol) and **14tfib** (100.3 mg, 0.250 mmol) was dissolved in a solvent mixture of 2.0 mL diethyl ether and 2.0 mL petroleum ether and left to crystallize at room temperature.

Crystal bulk of **(BM)(135tfib)**$_2$

A mixture of **BM** (27.4 mg, 0.100 mmol) and **135tfib** (102.0 mg, 0.200 mmol) was dissolved in a solvent mixture of 1.0 mL methanol and 1.0 mL acetonitrile and left to crystallize at room temperature.

Crystal bulk of **(BP)(14tfib)**

A 100 µL aliquot of the **BP** solution (0.100 mmol) was diluted in 2 mL of tetrahydrofuran, followed by the dissolution of 40.1 mg of **14tfib** (0.100 mmol). The solution was then left to crystallize at room temperature.

Crystal bulk of **(BP)(135tfib)**$_2$

A 100 µL aliquot of the **BP** solution (0.100 mmol) was diluted in a solvent mixture of 1.0 mL methanol and 1.0 mL acetonitrile, followed by the dissolution of 102.0 mg of **135tfib** (0.200 mmol). The solution was then left to crystallize at room temperature.

Crystal bulk of **(AM)(14tfib)**

A 100 µL aliquot of the **AM** solution (0.100 mmol) was diluted in 2 mL of tetrahydrofuran, followed by the dissolution of 40.1 mg of **14tfib** (0.100 mmol). The solution was then left to crystallize at room temperature.

Crystal bulk of **(AM)(135tfib)**$_2$

A 100 µL aliquot of the **AM** solution (0.100 mmol) was diluted in a solvent mixture of 1.0 mL methanol and 1.0 mL acetonitrile, followed by the dissolution of 102.0 mg of **135tfib** (0.200 mmol). The solution was then left to crystallize at room temperature.

Crystal bulk of **(AP)(14tfib)**

A 100 µL aliquot of the **AP** solution (0.100 mmol) was diluted in a solvent mixture of 2.0 mL diethyl ether and 2.0 mL petroleum ether, followed by the dissolution of 40.1 mg of **14tfib** (0.100 mmol). The solution was then left to crystallize at room temperature.

Crystal bulk of **(AP)(135tfib)**

A 100 µL aliquot of the **AP** solution (0.100 mmol) was diluted in 2 mL of tetrahydrofuran, followed by the dissolution of 51.0 mg of **135tfib** (0.100 mmol). The solution was then left to crystallize at room temperature.

THERMAL ANALYSIS

TG-DSC measurements were performed on a Mettler-Toledo *TG/DSC 3+* instrument. The samples were placed in sealed aluminium pans (40 μL) with a pinhole made in the top cover, and heated in flowing nitrogen (50 mL min^{-1}) from 25 °C to 300 °C (25 °C to 500 °C for **BM**) at a rate of 10 °C min^{-1}. Data collection and analysis were performed using the program package STAR^{e} Software 15.00.[1]

INFRARED SPECTROSCOPY

Infrared spectroscopic analysis in the far-infrared area (range 800-110 cm^{-1}) was performed on a *Nicolet iS50* FTIR spectrometer with an ATR module, using a solid-substrate beam splitter. Spectra of the prepared crystal bulk samples were recorded at room temperature as a combination of 32 scans, and with a spectral resolution of 4 cm^{-1}. IR spectra were collected, processed and visualized by the *OMNIC™ Specta Software*.[3]

POWDER X-RAY DIFFRACTION EXPERIMENTS

PXRD experiments were performed on a Malvern PANalytical *Aeris* X-ray diffractometer with Cu$K\alpha 1$ (1.54056 Å) radiation at 15 mA and 40 kV. The scattered intensities were measured with a line (1D) detector. The angular range was from 5 to 40° (2θ) with an interpolated step size of 0.00543322°. Data analysis was performed using the program *Data Viewer*.[3]

SINGLE-CRYSTAL X-RAY DIFFRACTION EXPERIMENTS

The crystal and molecular structures of the prepared cocrystals were determined by single crystal X-ray diffraction. Details of data collection and crystal structure refinement are listed in Table S1, S2, S3 and S4. Diffraction measurements were made on and Rigaku Synergy XtaLAB X-ray diffractometer with graphite-monochromated MoKα (λ = 0.71073Å) radiation. The data sets were collected using the ω scan mode over the 2θ range up to 64°. The *CrysAlisPro* program package was employed for data collection, cell refinement, and data reduction.[4] The structures were solved by direct methods and refined using the *SHELXS*, *SHELXT*, and *SHELXL* programs, respectively.[5,6] The structural refinement was performed on F^2 using all data. Hydrogen atoms were placed in calculated positions and treated as riding on their parent atoms All calculations were performed using the *WINGX* crystallographic suite of programs.[7] The molecular structures of compounds and their molecular packing projections were prepared by *Mercury*.[8]

COMPUTATION DETAILS

All calculations were performed using Gaussian 16 software package[9]. Geometry optimizations were performed using M062X/def2-tzvp level of theory,[10] with ultrafine integration grid (99 radial shells and 590 points per shell) starting from acceptor molecule geometries which have been found in corresponding crystal structure. Harmonic frequency calculations were performed on the optimized geometries to ensure the success of each geometry optimization. The figures were prepared using GaussView.[11]

References

1. STARe Evaluation Software Version 15.00, Mettler–Toledo GmbH, 2016.
2. Data Viewer Version 1.9a, PANalytical B.V. Amelo, The Netherlands, 2018.
3. Omnic Specta Software 9.9.549, Thermo Fisher Scientific, 2018.
4. Rigaku Oxford Diffraction, Gemini CCD system, CrysAlis Pro software, Version 171.41.93a, 2020.
5. (a) G. M. Sheldrick, *Acta Cryst. A*, 2008, **64**, 112–122; (b) G. M. Sheldrick, *Acta Cryst. C*, 2015, **71**, 3–8.
6. G. M. Sheldrick, *Acta Cryst. A*, 2015, **71**, 3–8.
7. L. J. Farrugia, *J. Appl. Cryst.*, 2012, **45**, 849–854.
8. C. F. Macrae, I. J. Bruno, J. A. Chisholm, P. R. Edgington, P. McCabe, E. Pidcock, L. Rodriguez-Monge, R. Taylor, J. v. d. Streek and P. A. Wood, *J. Appl. Crystallogr.* **2008**, 41, 466.
9. Gaussian 16, Revision C.01, Frisch, M. J.; Trucks, G. W.; Schlegel, H. B.; Scuseria, G. E.; Robb, M. A.; Cheeseman, J. R.; Scalmani, G.; Barone, V.; Petersson, G. A.; Nakatsuji, H.; Li, X.; Caricato, M.; Marenich, A. V.; Bloino, J.; Janesko, B. G.; Gomperts, R.; Mennucci, B.; Hratchian, H. P.; Ortiz, J. V.; Izmaylov, A. F.; Sonnenberg, J. L.; Williams-Young, D.; Ding, F.; Lipparini, F.; Egidi, F.; Goings, J.; Peng, B.; Petrone, A.; Henderson, T.; Ranasinghe, D.; Zakrzewski, V. G.; Gao, J.; Rega, N.; Zheng, G.; Liang, W.; Hada, M.; Ehara, M.; Toyota, K.; Fukuda, R.; Hasegawa, J.; Ishida, M.; Nakajima, T.; Honda, Y.; Kitao, O.; Nakai, H.; Vreven, T.; Throssell, K.; Montgomery, J. A., Jr.; Peralta, J. E.; Ogliaro, F.; Bearpark, M. J.; Heyd, J. J.; Brothers, E. N.; Kudin, K. N.; Staroverov, V. N.; Keith, T. A.; Kobayashi, R.; Normand, J.; Raghavachari, K.; Rendell, A. P.; Burant, J. C.; Iyengar, S. S.; Tomasi, J.; Cossi, M.; Millam, J. M.; Klene, M.; Adamo, C.; Cammi, R.; Ochterski, J. W.; Martin, R. L.; Morokuma, K.; Farkas, O.; Foresman, J. B.; Fox, D. J. Gaussian, Inc., Wallingford CT, 2016.
10. Zhao, Y.; Truhlar, D.G. The M06 suite of density functionals for main group thermochemistry, thermochemical kinetics, noncovalent interactions, excited states, and transition elements: Two new functionals and systematic testing of four M06-class functionals and 12 other functionals. *Theor. Chem. Acc.*, **2008**, 120, 215–241.
11. GaussView, Version 5.1; Dennington, R.; Keith, T.A.; Millam, J.M. (Eds.) Semichem Inc.: Shawnee, KS, USA, 2008.

Table S1. Crystal data and refinement details for the prepared compounds.

	**BM**
Molecular formula	$C_{16}H_{22}N_2O_2$
M_r	274.35
Crystal system	triclinic
Space group	$P\,-1$
Crystal data:	
a / Å	6.8079(2)
b / Å	7.3685(3)
c / Å	15.5223(6)
α / °	82.051(3)
β / °	82.092(3)
γ / °	85.271(3)
V / Å^3	762.25(5)
Z	2
D_{calc} / g cm^{-3}	1.195
λ(MoK_α) / Å	0.71073
T / K	294.98
Crystal size / mm^3	0.59 x 0.54 x 0.33
μ / mm^{-1}	0.079
F(000)	296.0
Refl. collected/unique	5094/3326
Parameters/restraints	183/0
$\Delta\rho_{max}$, $\Delta\rho_{min}$ / e Å^{-3}	0.219, –0.214
$R[F^2 > 4\sigma(F^2)]$	0.0520
w$R(F^2)$	0.1739
Goodness-of-fit, S	0.992

Table S1. continued

	**(BM)**$_2$**(14tfib)**$_5$	**(BM)(135tfib)**$_2$
Molecular formula	$(C_{16}H_{22}N_2O_2)_2(C_6F_4I_2)_5$	$(C_{16}H_{22}N_2O_2)(C_6F_3I_3)_2$
M_r	2558.01	1293.87
Crystal system	triclinic	triclinic
Space group	$P\,-1$	$P\,-1$
Crystal data:		
a / Å	10.0231(9)	9.4465(2)
b / Å	13.3635(9)	13.1951(3)
c / Å	15.3423(8)	15.4655(3)
α / °	86.631(5)	78.173(2)
β / °	86.708(6)	74.132(2)
γ / °	74.223(7)	85.622(2)
V / Å^3	1972.3(3)	1814.49(7)
Z	1	2
D_{calc} / g cm^{-3}	2.154	2.368
λ(MoK_α) / Å	0.71073	0.71073
T / K	295	294.98(12)
Crystal size / mm^3	0.36x0.26x0.15	0.50x 0.40 x 0.18
μ / mm^{-1}	4.020	5.195
F(000)	1186.0	1184.0
Refl. collected/unique	13150/7118	12307/7989
Parameters/restraints	452/0	399/0
$\Delta\rho_{max}$, $\Delta\rho_{min}$ / e Å^{-3}	1.647, –1.568	1.590, −1.476
$R[F^2 > 4\sigma(F^2)]$	0.0416	0.0513
w$R(F^2)$	0.130	0.1448
Goodness-of-fit, S	1.062	1.072

Table S1. continued

	**(BP)(14tfib)**	**(BP)(135tfib)$_2$**
Molecular formula	$(C_{16}H_{23}N_3O)(C_6F_4I_2)$	$(C_{16}H_{23}N_3O)(C_6F_3I_3)_2$
M_r	675.23	1292.89
Crystal system	triclinic	triclinic
Space group	$P\,{-1}$	$P\,{-1}$
Crystal data:		
a / Å	10.8461(4)	9.4086(2)
b / Å	11.4690(3)	16.1261(3)
c / Å	11.5494(4)	24.6535(4)
α / °	68.838(3)	71.160(2)
β / °	69.003(3)	85.5070(10)
γ / °	75.024(3)	83.099(2)
V / Å^3	1237.01(8)	3511.37(12)
Z	2	4
D_{calc} / g cm^{-3}	1.813	2.446
$\lambda(\mathrm{Mo}K_\alpha)$ / Å	0.71073	0.71073
T / K	295	295
Crystal size / mm^3	0.61 x 0.54 x 0.40	0.63x 0.46 x 0.16
μ / mm^{-1}	2.591	5.367
F(000)	652	2368
Refl. collected/unique	8341/5826	15284/12664
Parameters/restraints	295/0	801/2
$\Delta\rho_{max}$, $\Delta\rho_{min}$ / e Å^{-3}	0.928,–1.104	3.792,–2.470
$R[F^2 > 4\sigma(F^2)]$	0.0401	0.0758
w$R(F^2)$	0.0979	0.2233
Goodness-of-fit, S	0.935	1.074

Table S1. continued

	**(AM)(14tfib)**	**(AM)(135tfib)**$_2$
Molecular formula	$(C_{11}H_{20}N_2O_2)(C_6F_4I_2)$	$(C_{11}H_{20}N_2O_2)(C_6F_3I_3)_2$
M_r	614.15	1231.81
Crystal system	triclinic	monoclinic
Space group	$P\,-1$	$P\,2_1/c$
Crystal data:		
a / Å	10.0384(4)	28.4806(9)
b / Å	10.3499(3)	9.4163(2)
c / Å	10.7293(4)	25.4977(6)
α / °	88.165(3)	90
β / °	88.420(3)	105.184(3)
γ / °	77.033(3)	90
V / Å^3	1085.52(7)	6599.3(3)
Z	2	8
D_{calc} / g cm^{-3}	1.879	2.480
$\lambda(MoK_\alpha)$ / Å	0.71073	0.71073
T / K	295.01(18)	301(3)
Crystal size / mm^3	0.58 x 0.55 x 0.26	0.40 x 0.19 x 0.08
μ / mm^{-1}	2.944	5.706
F(000)	588	4480
Refl. collected/unique	7344/4447	14384/10077
Parameters/restraints	265/0	726/0
$\Delta\rho_{max}$, $\Delta\rho_{min}$ / e Å^{-3}	0.809, –0.643	0.809, –0.643
$R[F^2 > 4\sigma(F^2)]$	0.0424	0.0443
w$R(F^2)$	0.1270	0.1091
Goodness-of-fit, S	0.979	1.053

Table S1. continued

	**(AP)(14tfib)**	**(AP)(135tfib)**
Molecular formula	$(C_{11}H_{21}N_3O)(C_6F_4I_2)$	$(C_{11}H_{21}N_3O)(C_6F_3I_3)$
M_r	613.17	721.07
Crystal system	triclinic	triclinic
Space group	$P\,-1$	$P\,-1$
Crystal data:		
a / Å	9.4321(5)	9.2985(2)
b / Å	10.3287(5)	10.7712(3)
c / Å	12.6238(6)	12.4997(4)
α / °	95.489(4)	106.777(3)
β / °	111.373(5)	103.116(2)
γ / °	94.174(4)	98.159(2)
V / Å^3	1132.28(10)	1138.11(6)
Z	2	2
D_{calc} / g cm^{-3}	1.798	2.104
$\lambda(\mathrm{Mo}K_\alpha)$ / Å	0.71073	0.71073
T / K	295.01(18)	294.99(10)
Crystal size / mm^3	0.55 x 0.47 x 0.19	0.49 x 0.38 x 0.14
μ / mm^{-1}	2.820	4.155
$F(000)$	588	676
Refl. collected/unique	4939/3158	7669/5336
Parameters/restraints	260/0	250/0
$\Delta\rho_{max}$, $\Delta\rho_{min}$ / e Å^{-3}	1.454, –0.792	0.627, –1.279
$R[F^2 > 4\sigma(F^2)]$	0.0583	0.0364
$wR(F^2)$	0.1774	0.0911
Goodness-of-fit, S	1.032	0.945

Table S2. Melting point and decomposition signal onset temperatures, t_E

cocrystal	t_E/°C
**(BM)**$_2$**(14tfib)**$_5$	64.9
**(BM)(135tfib)**$_2$	128.5
**(BP)(14tfib)**	128.3[a]
**(BP)(135tfib)**$_2$	125.2
**(AM)(14tfib)**	74.7
**(AM)(135tfib)**$_2$	110.7
**(AP)(14tfib)**	110.0
**(AP)(135tfib)**	92.7

[a] decomposition temperature

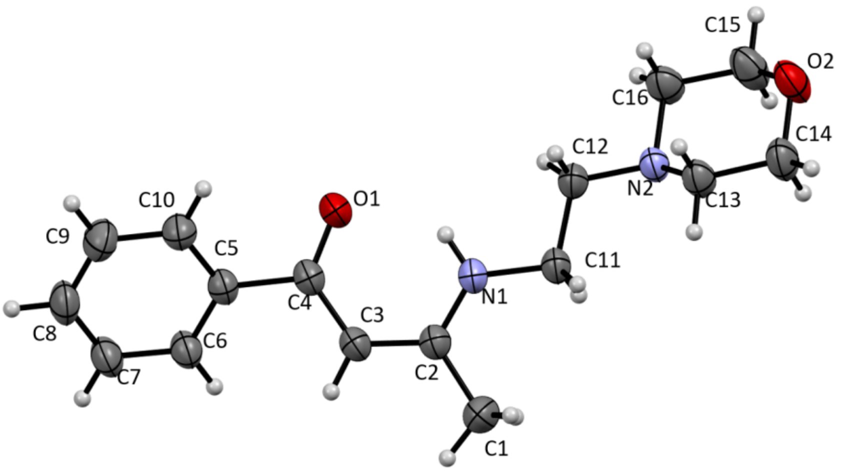

Figure S1. Partial molecular structure of **BM** showing the atom-labelling scheme. Displacement ellipsoids are drawn at the 50 % probability level, and H atoms are shown as small spheres of arbitrary radius.

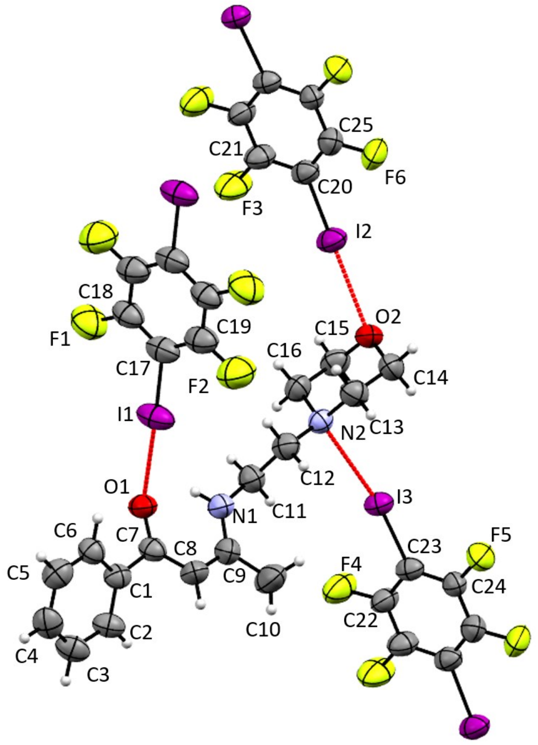

Figure S2. Partial molecular structure of (**BM**)$_2$(**14tfib**)$_5$ showing the atom-labelling scheme. Displacement ellipsoids are drawn at the 50 % probability level, and H atoms are shown as small spheres of arbitrary radius.

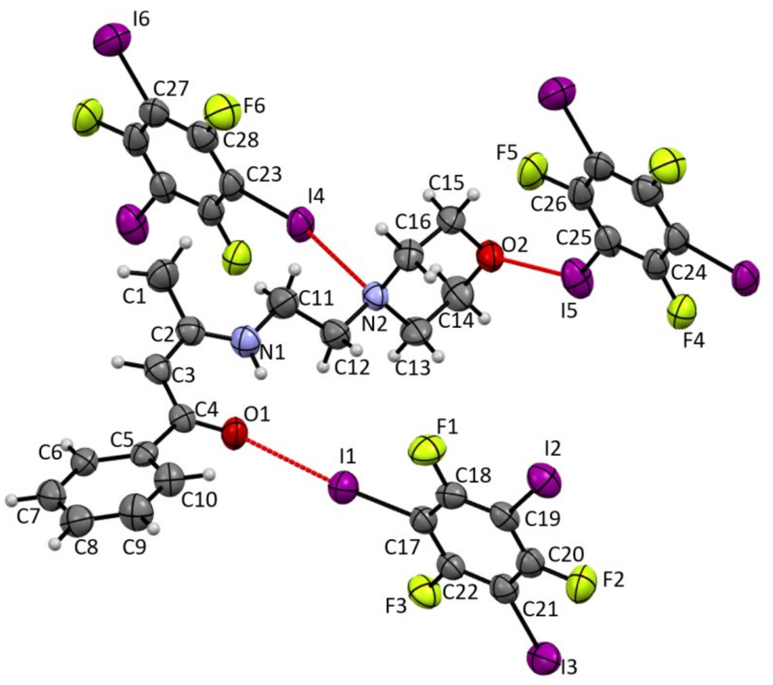

Figure S3. Partial molecular structure of (**BM**)(**135tfib**)$_2$ showing the atom-labelling scheme. Displacement ellipsoids are drawn at the 50 % probability level, and H atoms are shown as small spheres of arbitrary radius.

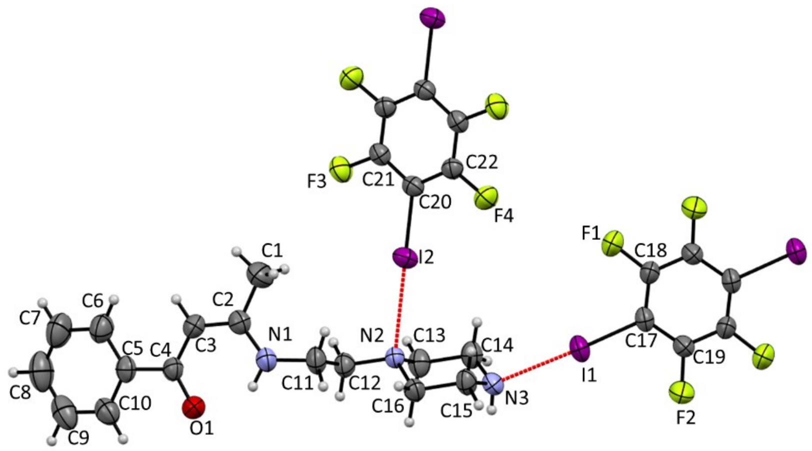

Figure S4. Partial molecular structure of **(BP)(14tfib)** showing the atom-labelling scheme. Displacement ellipsoids are drawn at the 50 % probability level, and H atoms are shown as small spheres of arbitrary radius.

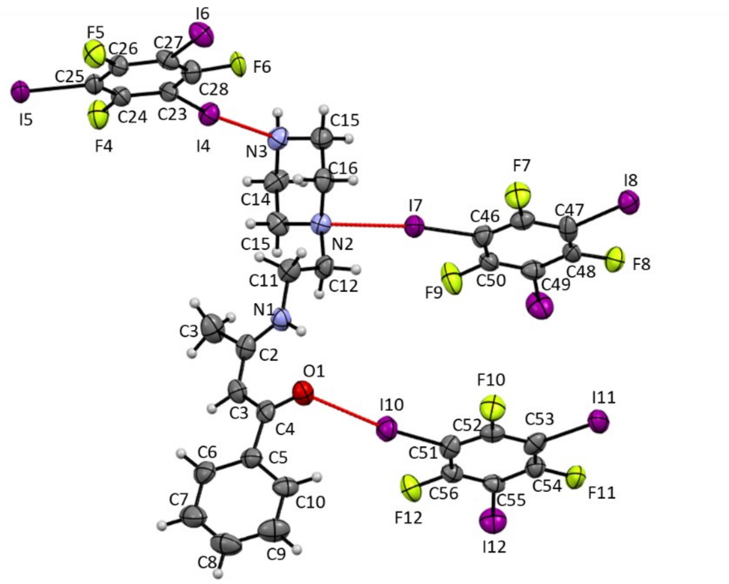

Figure S5. Partial molecular structure of (**BP**)(**135tfib**)$_2$ showing the atom-labelling scheme. Displacement ellipsoids are drawn at the 50 % probability level, and H atoms are shown as small spheres of arbitrary radius.

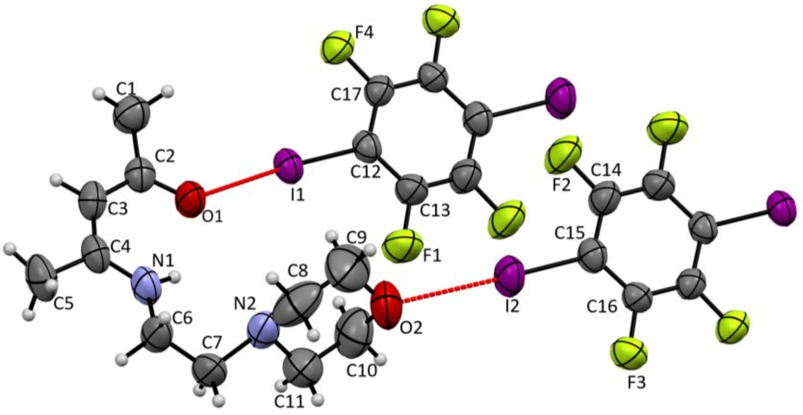

Figure S6. Partial molecular structure of (**AM**)(**14tfib**) showing the atom-labelling scheme. Displacement ellipsoids are drawn at the 50 % probability level, and H atoms are shown as small spheres of arbitrary radius.

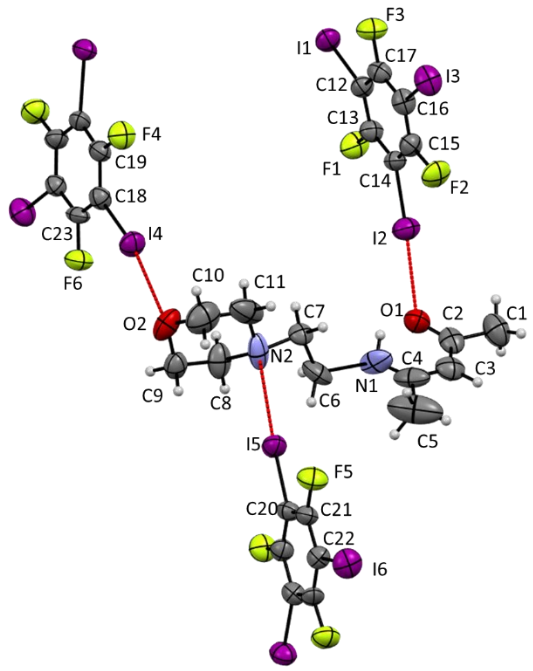

Figure S7. Partial molecular structure of (**AM**)(**135tfib**)$_2$ showing the atom-labelling scheme. Displacement ellipsoids are drawn at the 50 % probability level, and H atoms are shown as small spheres of arbitrary radius.

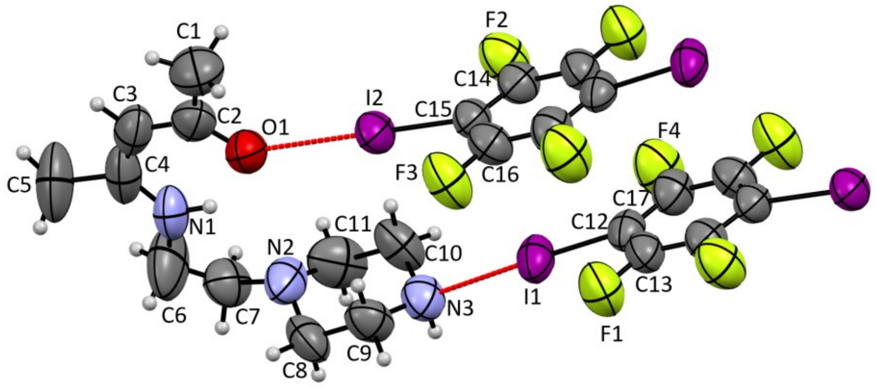

Figure S8. Partial molecular structure of (**AP**)(**14tfib**) showing the atom-labelling scheme. Displacement ellipsoids are drawn at the 50 % probability level, and H atoms are shown as small spheres of arbitrary radius.

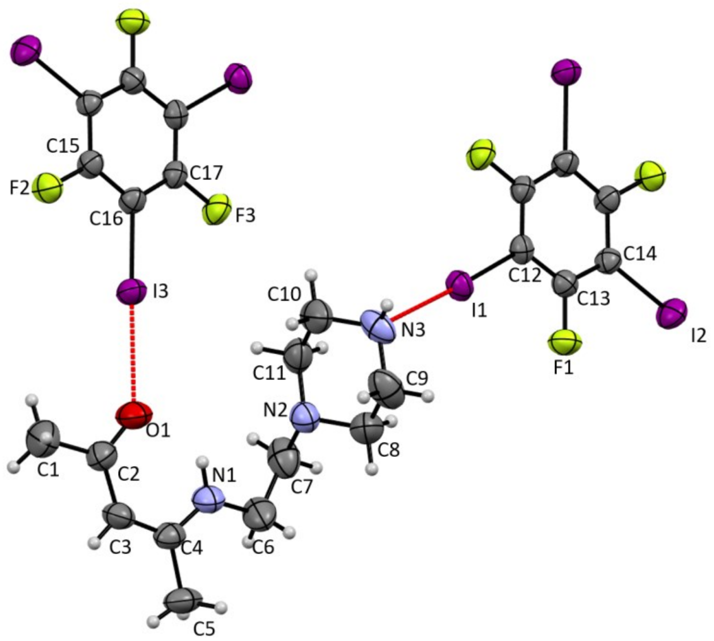

Figure S9. Partial molecular structure of (**AP**)(**135tfib**) showing the atom-labeling scheme. Displacement ellipsoids are drawn at the 50 % probability level, and H atoms are shown as small spheres of arbitrary radius.

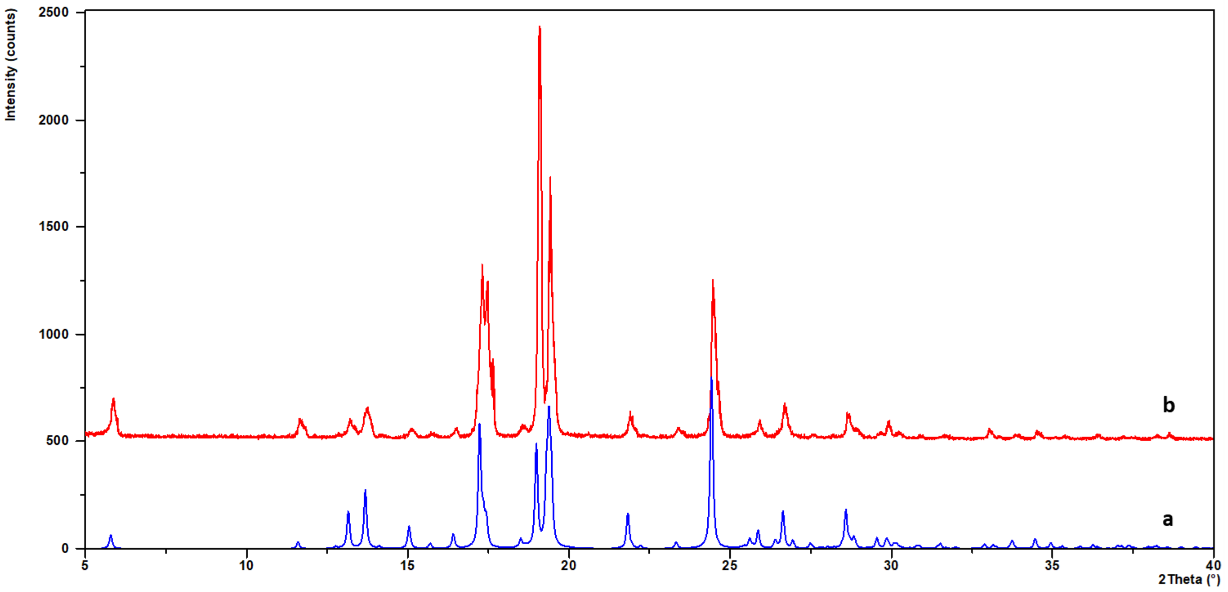

Figure S10. PXRD patterns of a) calculated pattern from **BM** single crystal data. b) product obtained by crystallization

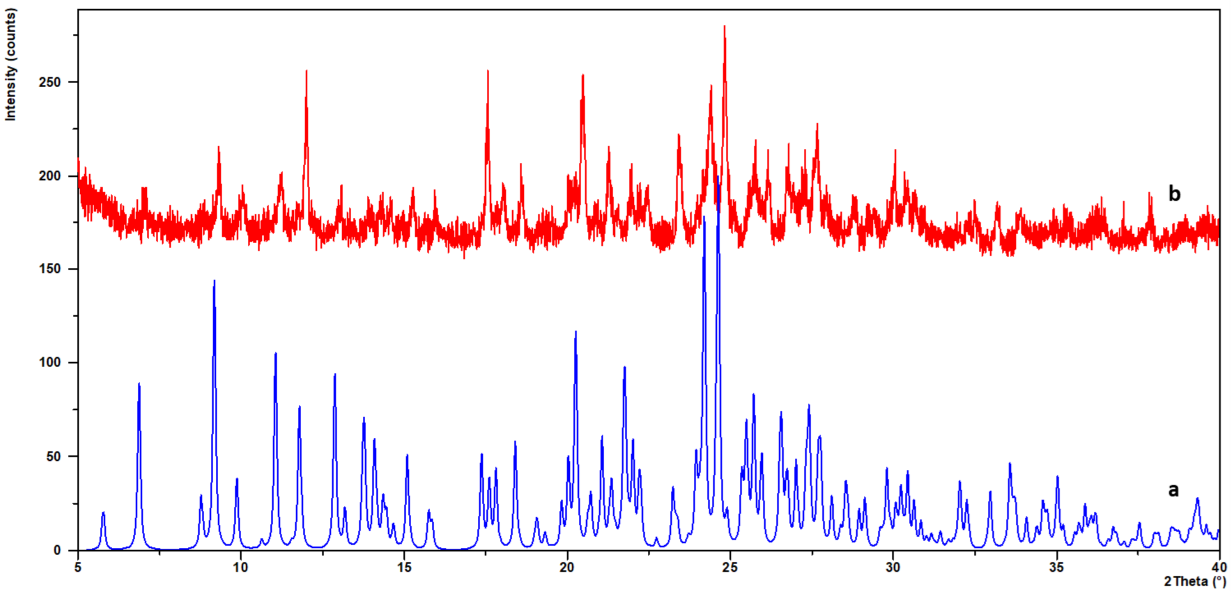

.

Figure S11. PXRD patterns of a) calculated pattern from (**BM**)$_2$(**14tfib**)$_5$ single crystal data. b) product obtained by crystallization

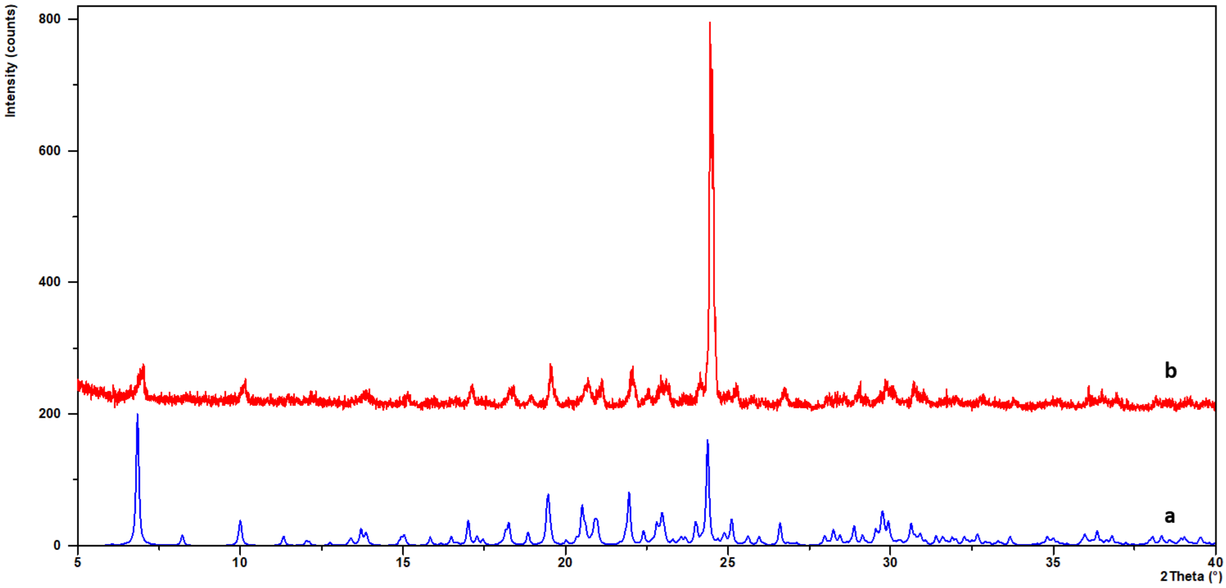

Figure S12. PXRD patterns of a) calculated pattern from (**BM**)(**135tfib**)$_2$ single crystal data. b) product obtained by crystallization

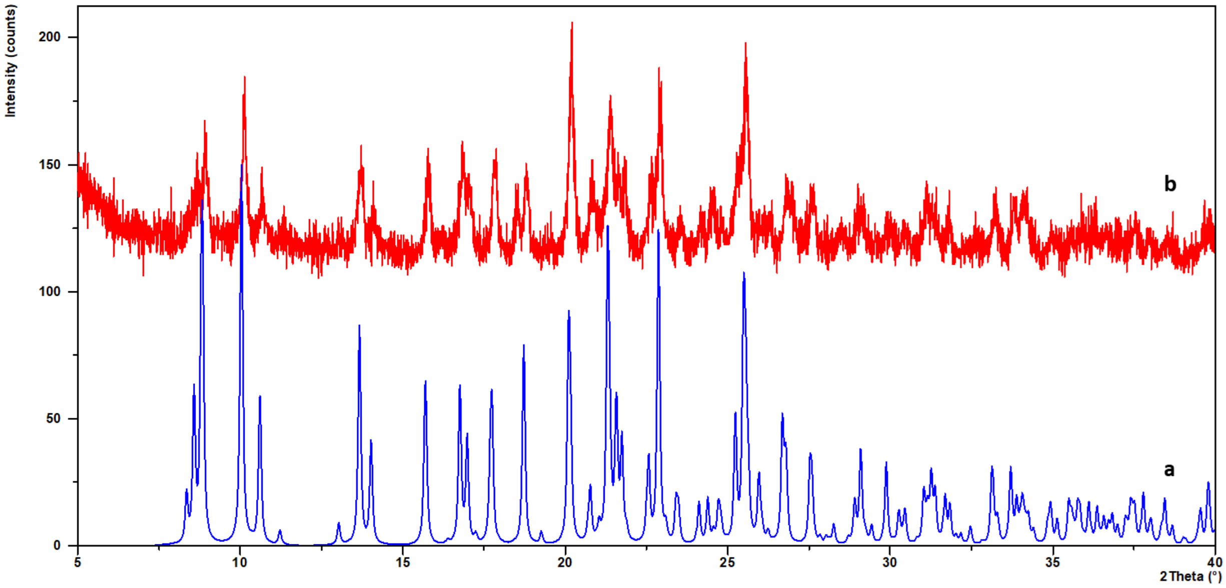

Figure S13. PXRD patterns of a) calculated pattern from (**BP**)(**14tfib**) single crystal data. b) product obtained by crystallization

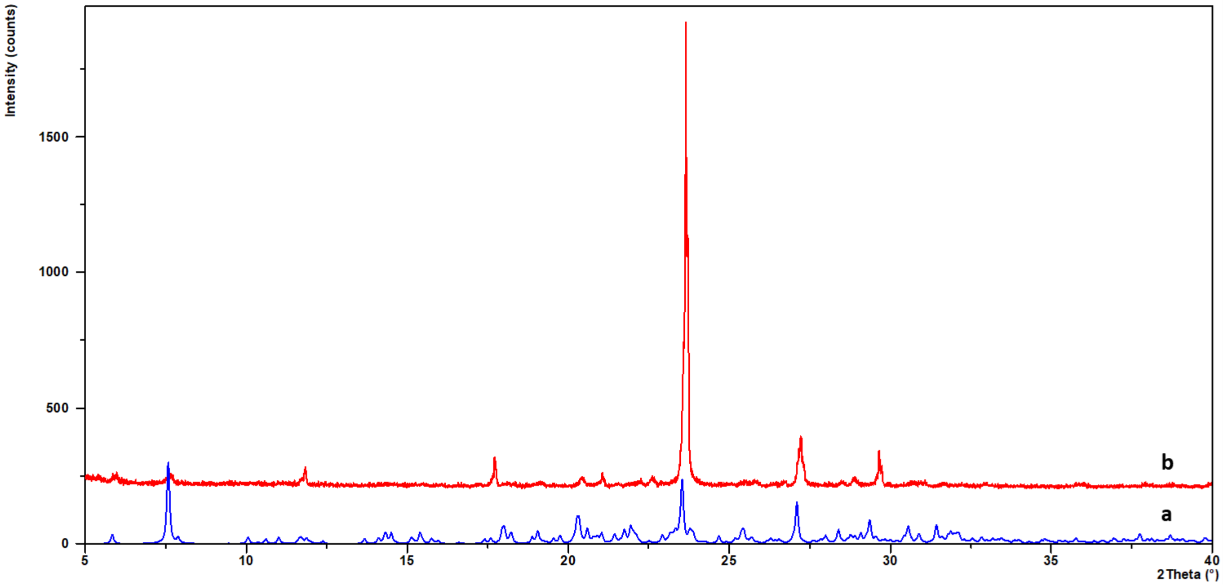

Figure S14. PXRD patterns of a) calculated pattern from (**BP**)(**135tfib**)$_2$ single crystal data. b) product obtained by crystallization

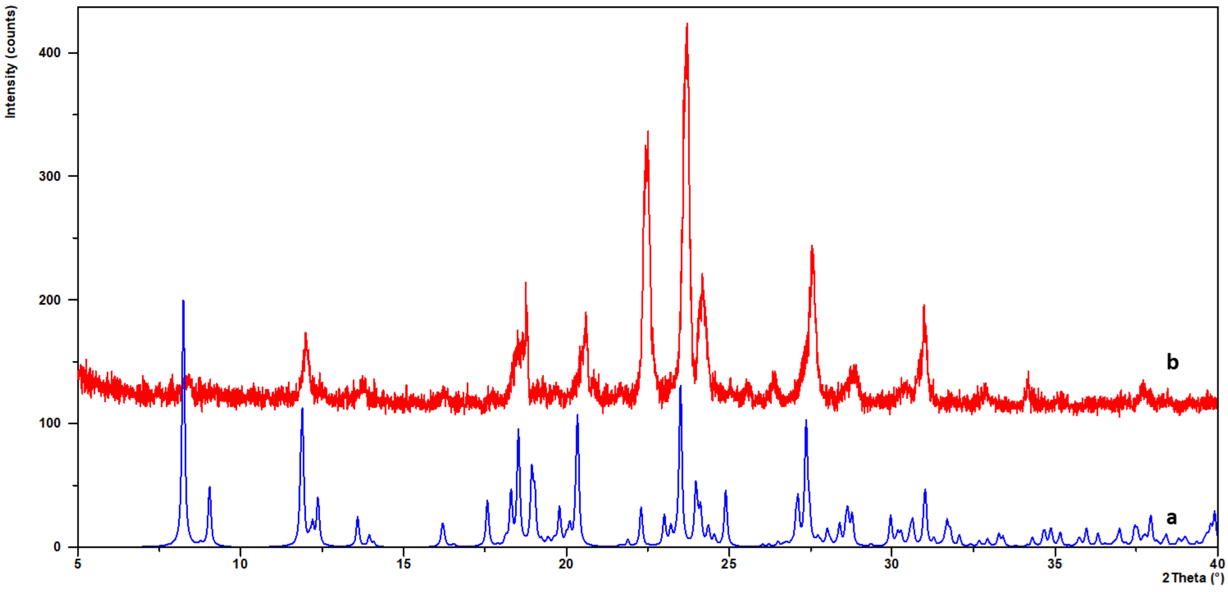

Figure S15. PXRD patterns of a) calculated pattern from (**AM**)(**14tfib**) single crystal data. b) product obtained by crystallization

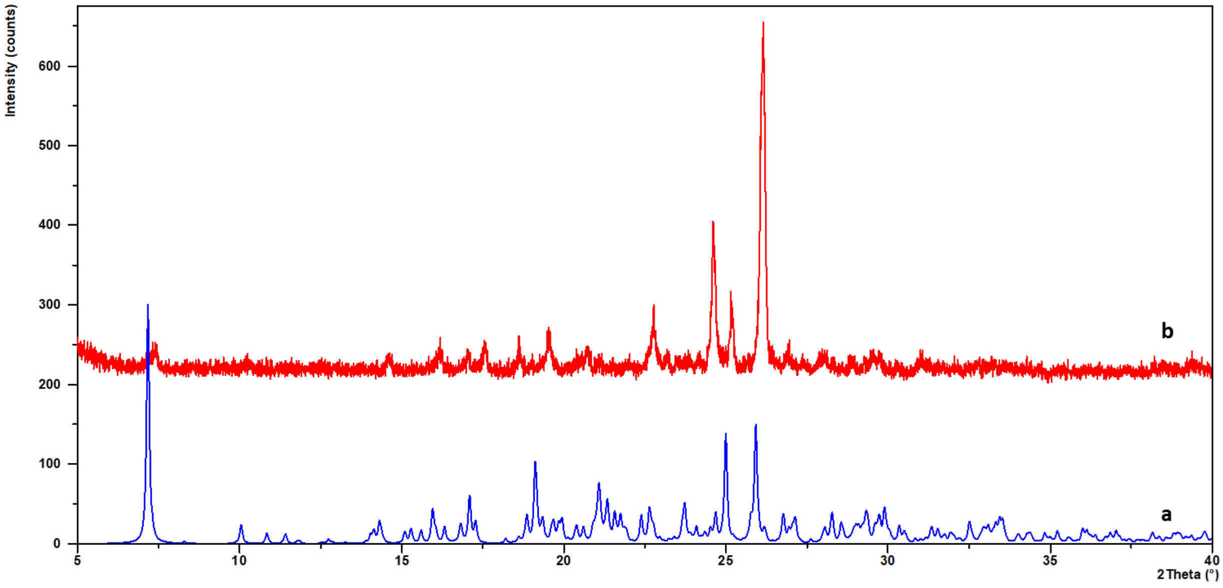

Figure S16. PXRD patterns of a) calculated pattern from (**AM**)(**135tfib**)$_2$ single crystal data. b) product obtained by crystallization

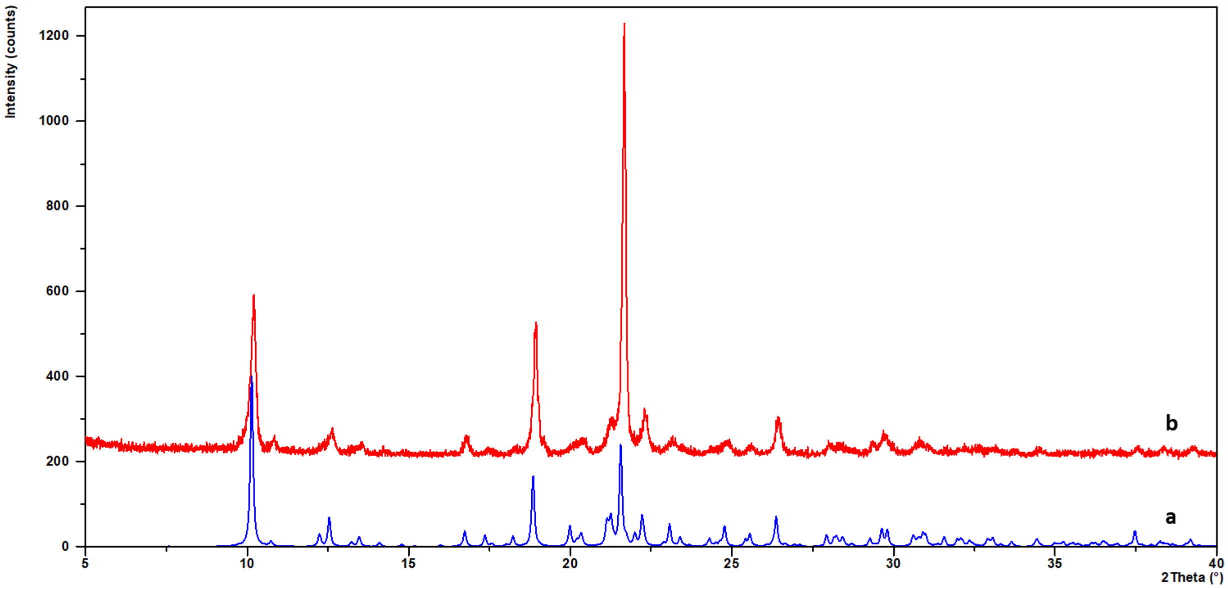

Figure S17. PXRD patterns of a) calculated pattern from (**AP**)(**14tfib**) single crystal data. b) product obtained by crystallization

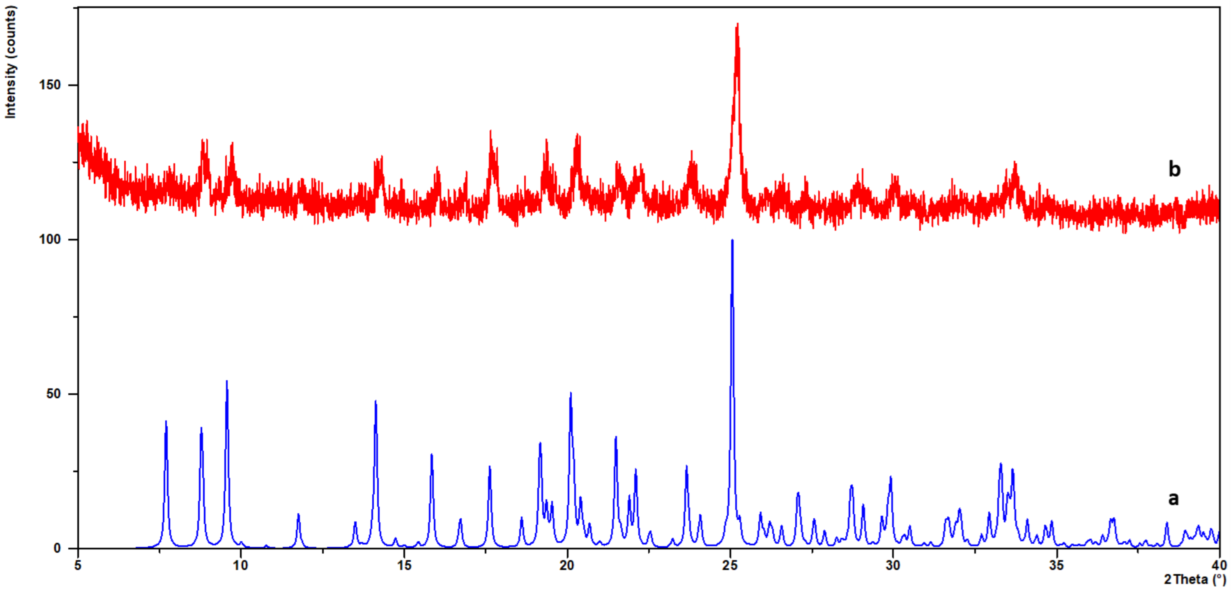

Figure S18. PXRD patterns of a) calculated pattern from (**AP**)(**135tfib**) single crystal data. b) product obtained by crystallization

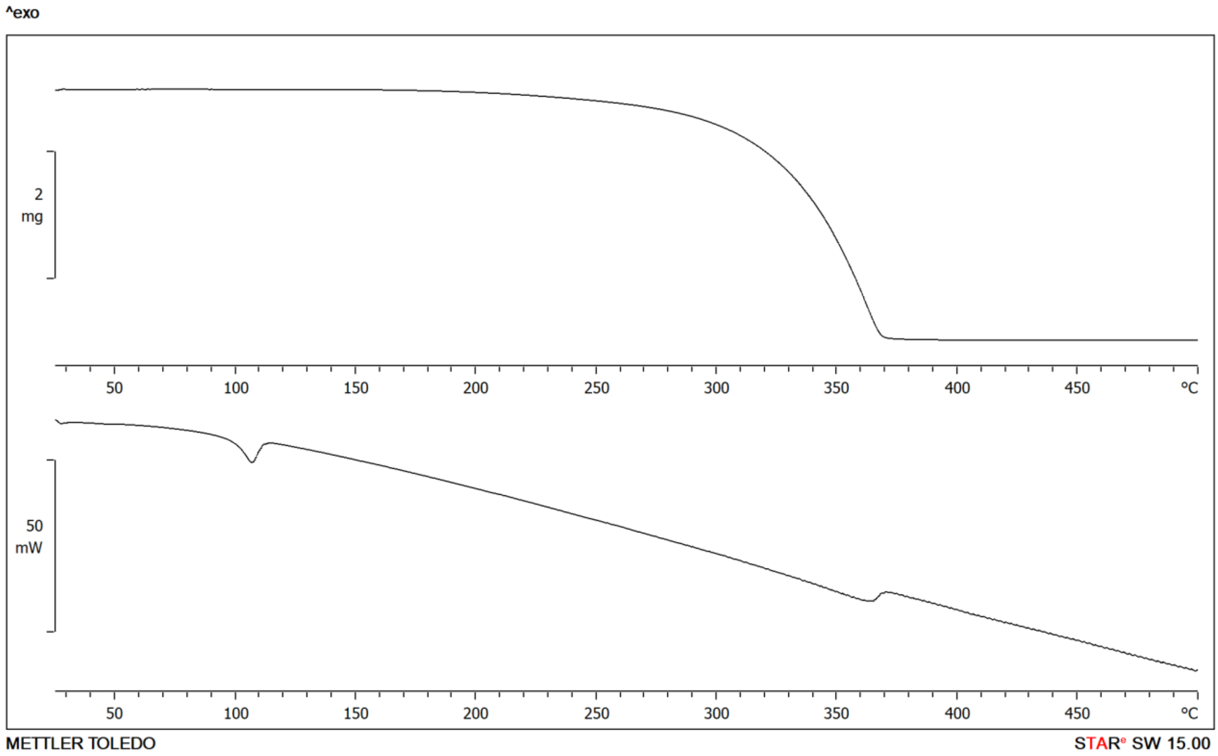

Figure S19. TG-DSC curve of **BM**

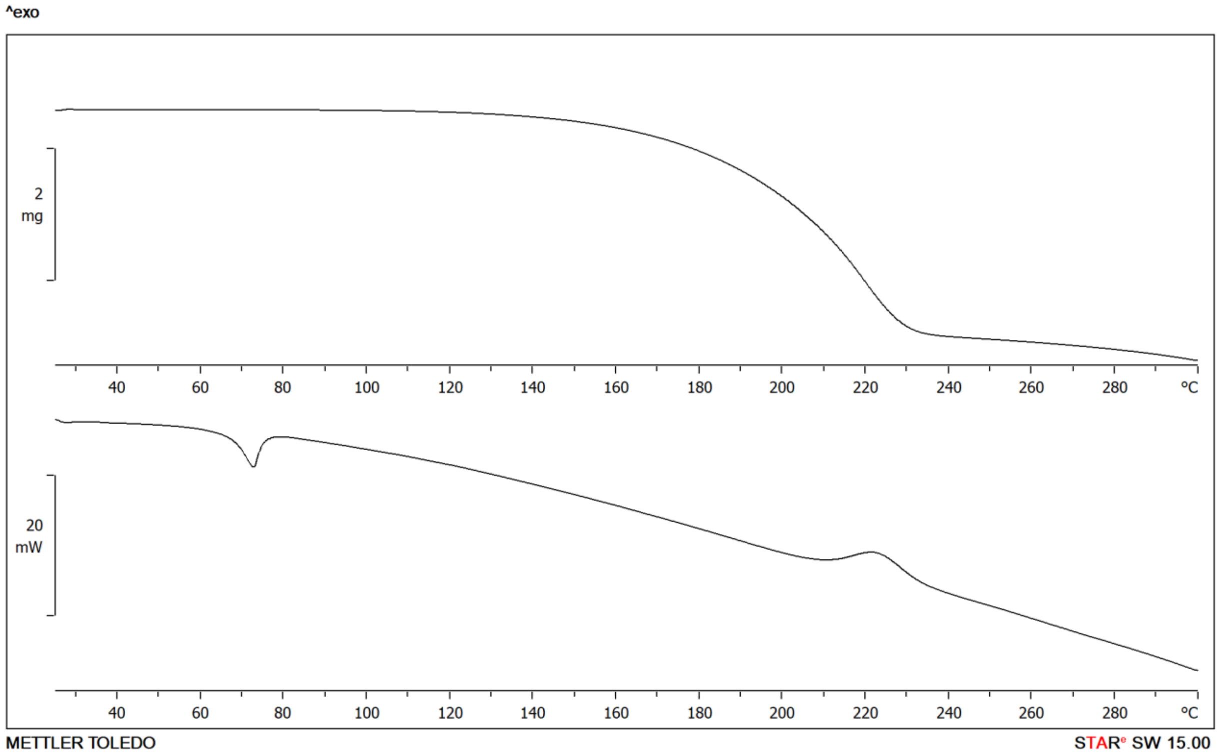

Figure S20. TG-DSC curve of (**BM**)$_2$(**14tfib**)$_5$.

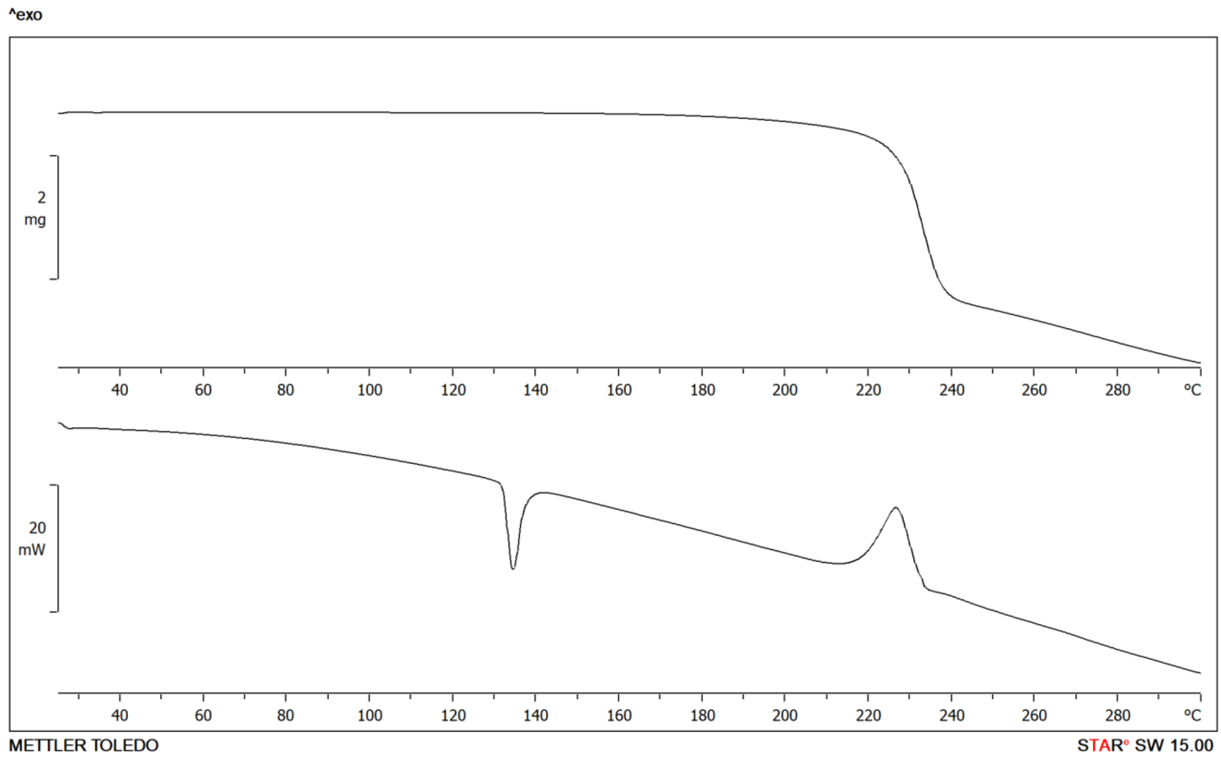

Figure S21. TG-DSC curve of (**BM**)(**135tfib**)$_2$

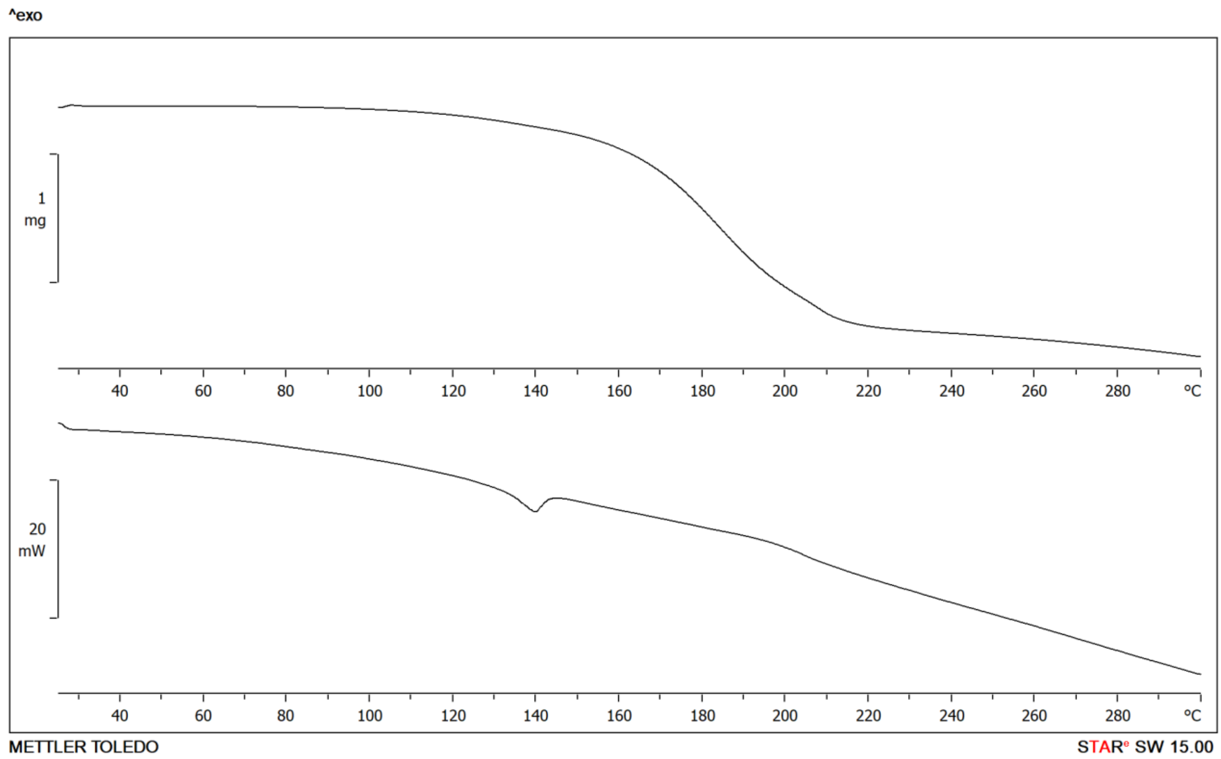

Figure S22. TG-DSC curve of **(BP)(14tfib).**

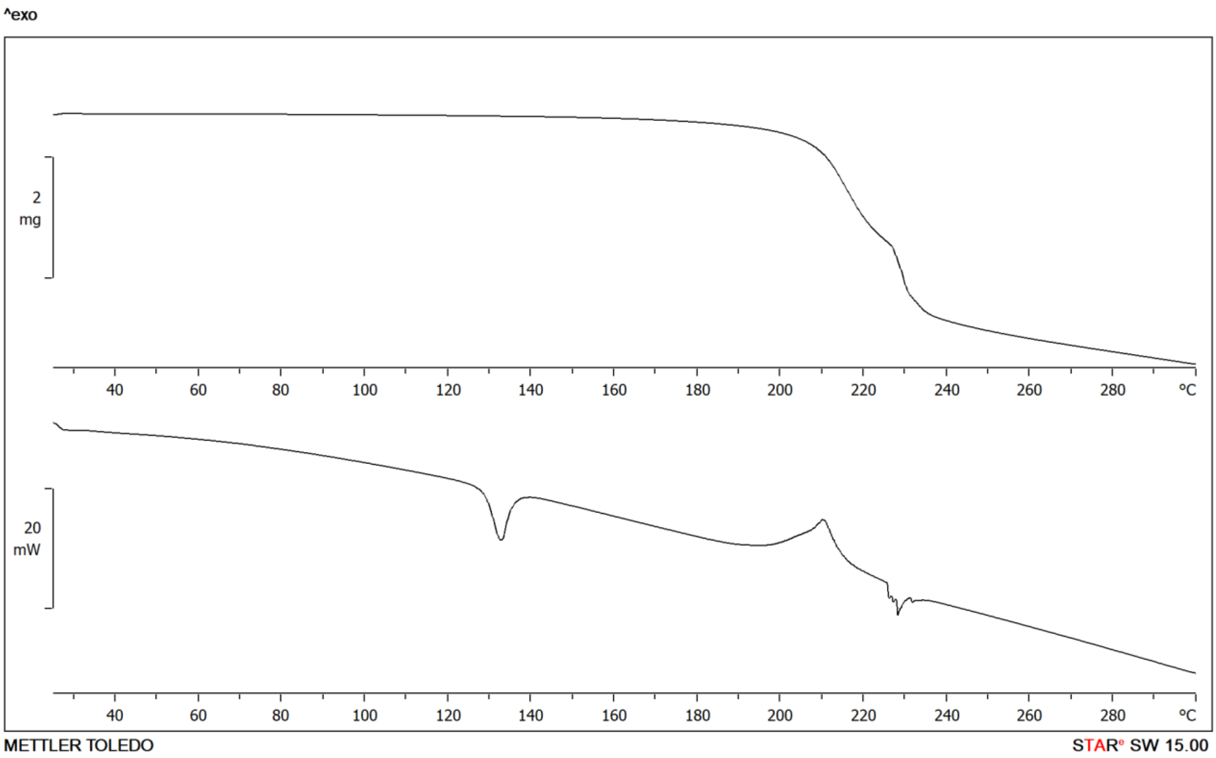

Figure S23. TG-DSC curve of **(BP)(135tfib)$_2$.**

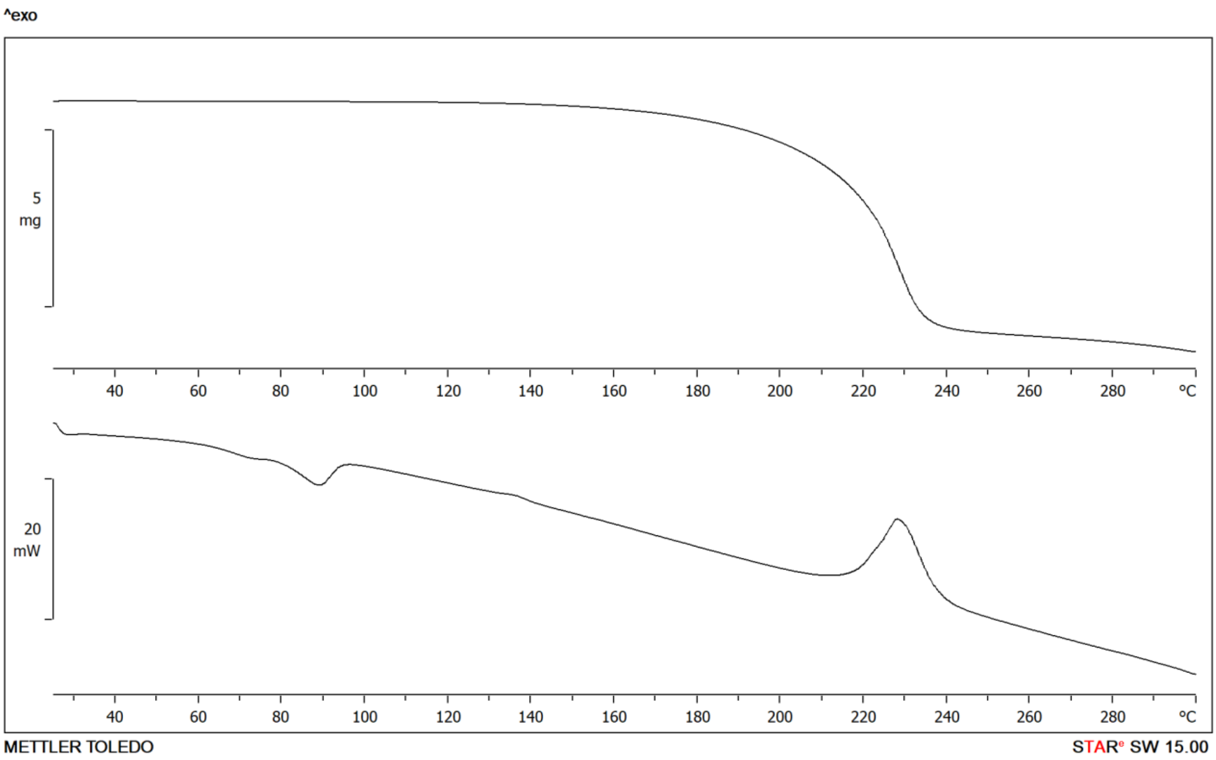

Figure S24. TG-DSC curve of (**AM**)(**14tfib**).

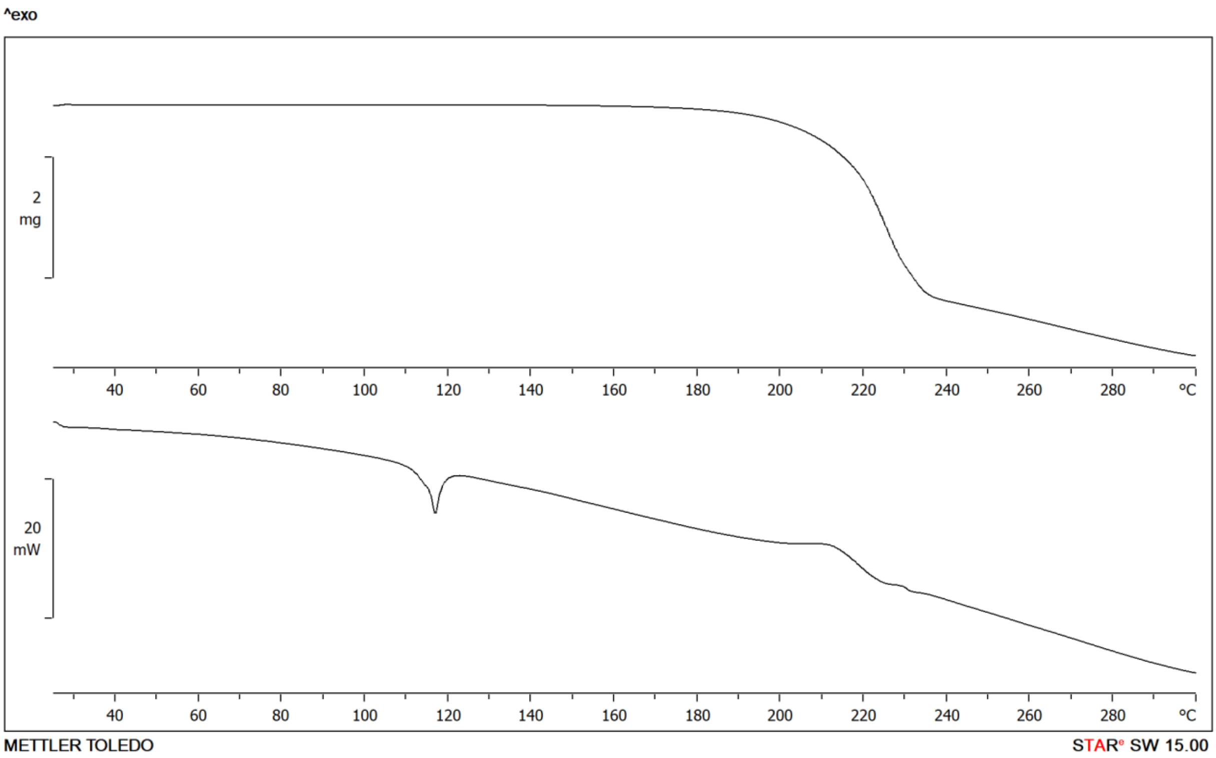

Figure S25. TG DSC curve of (**AM**)(**135tfib**)$_2$

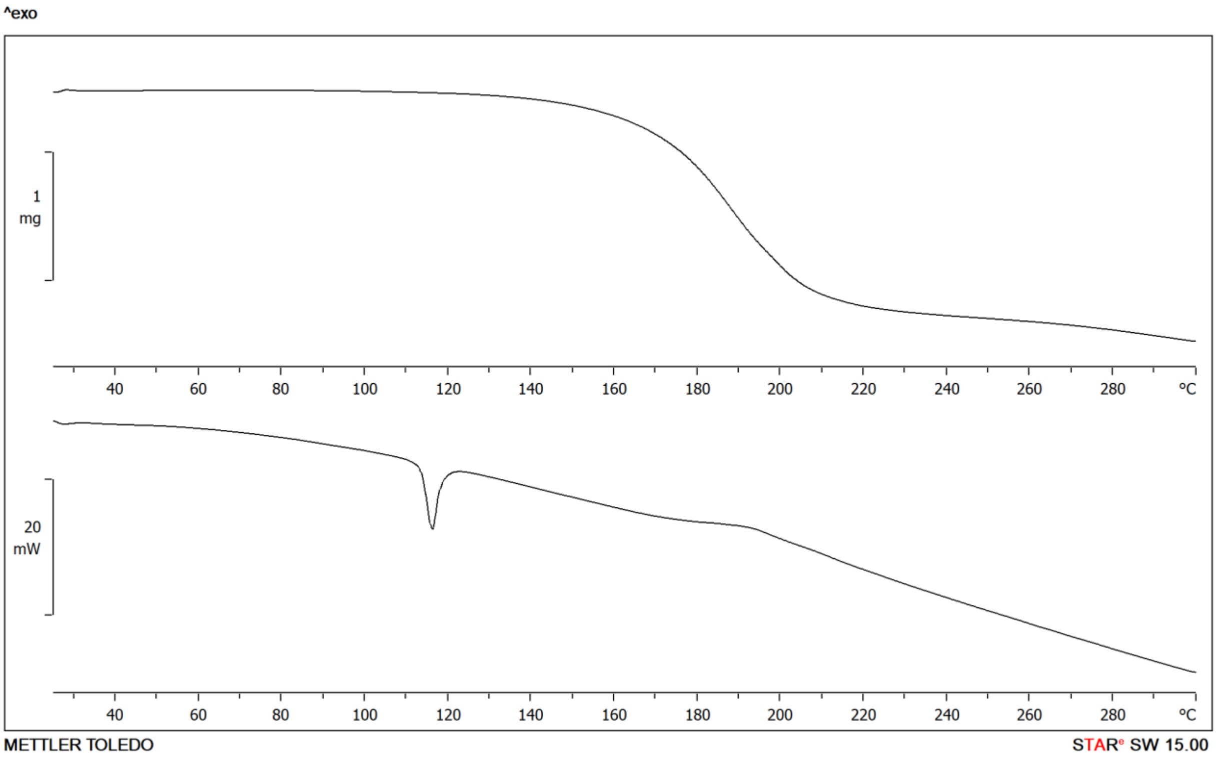

Figure S26. TG-DSC curve of (**AP**)(**14tfib**)

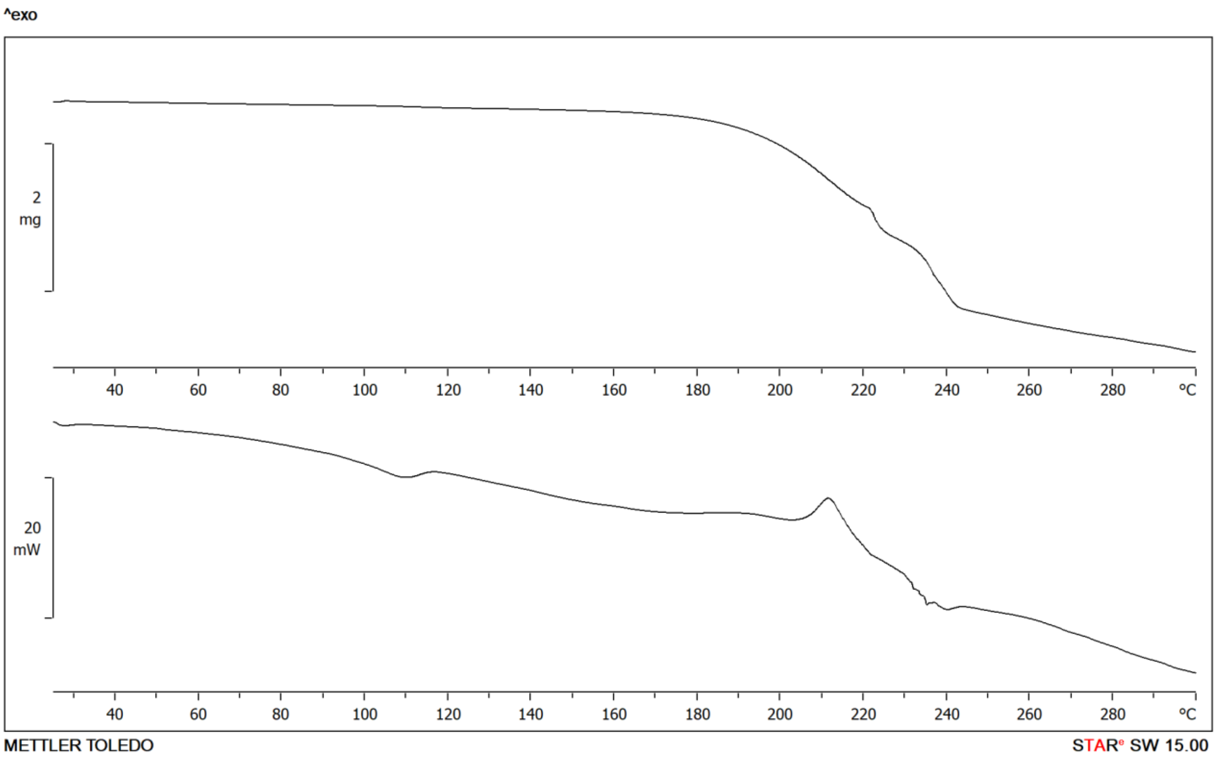

Figure S27. TG-DSC curve of (**AP**)(**135tfib**).

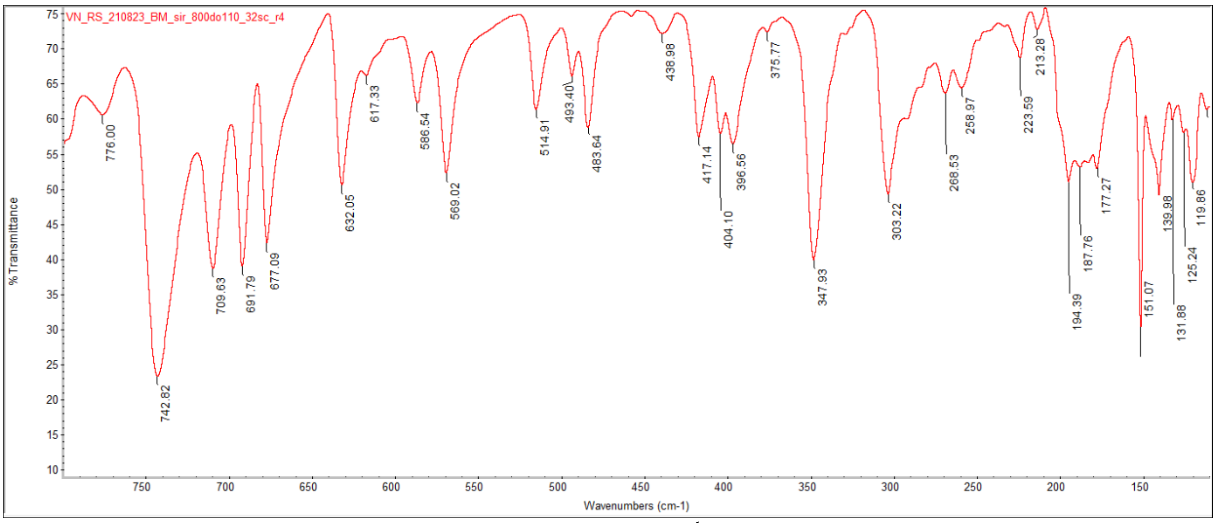

Figure S28. IR spectrum of **BM** from 110 to 800 cm^{-1}

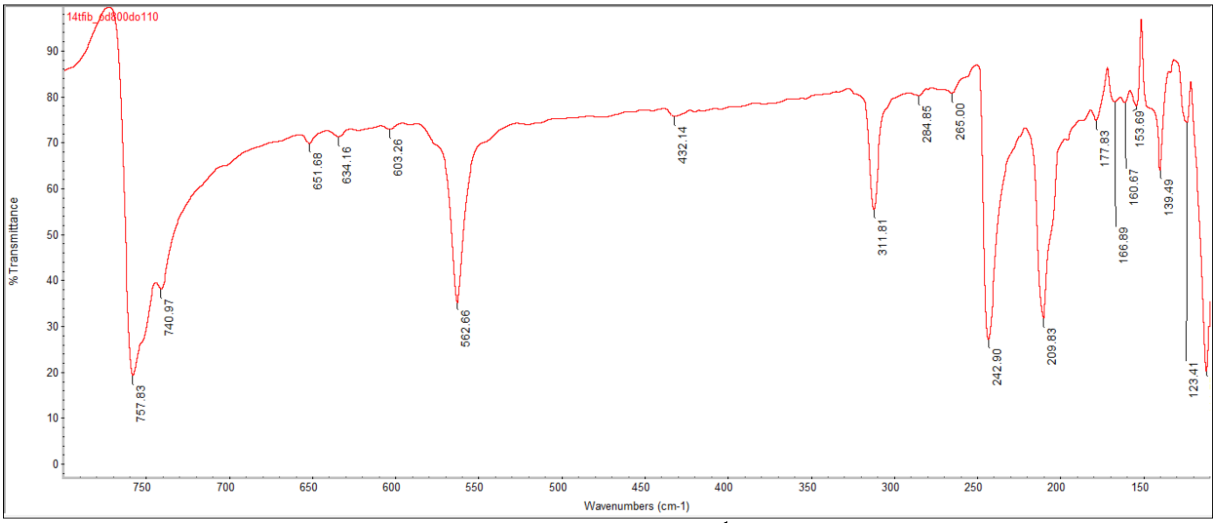

Figure S29. IR spectrum of **14tfib** from 110 to 800 cm^{-1}

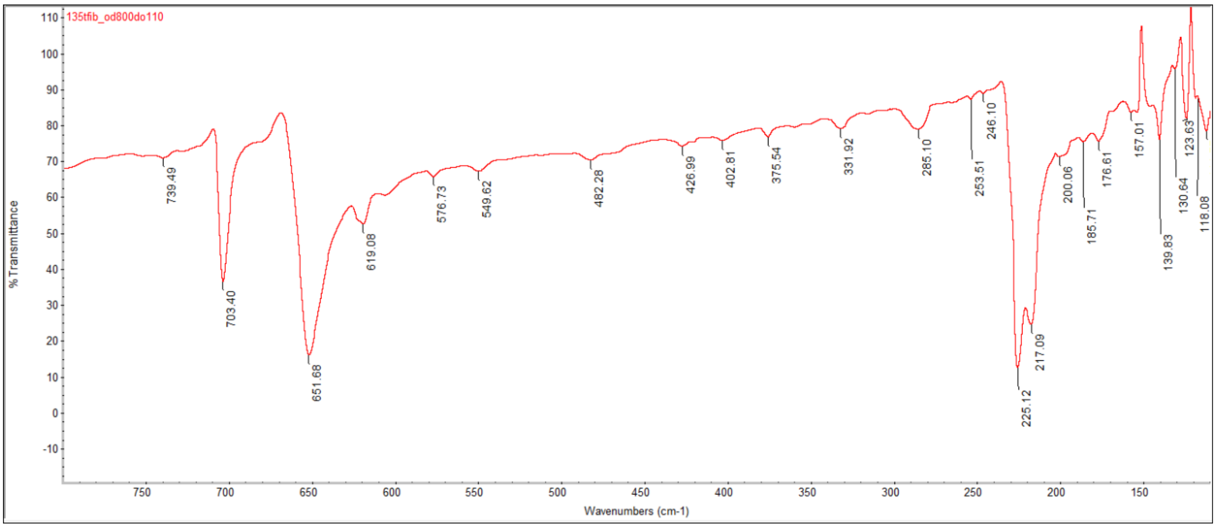

Figure S30. IR spectrum of **135tfib** from 110 to 800 cm^{-1}

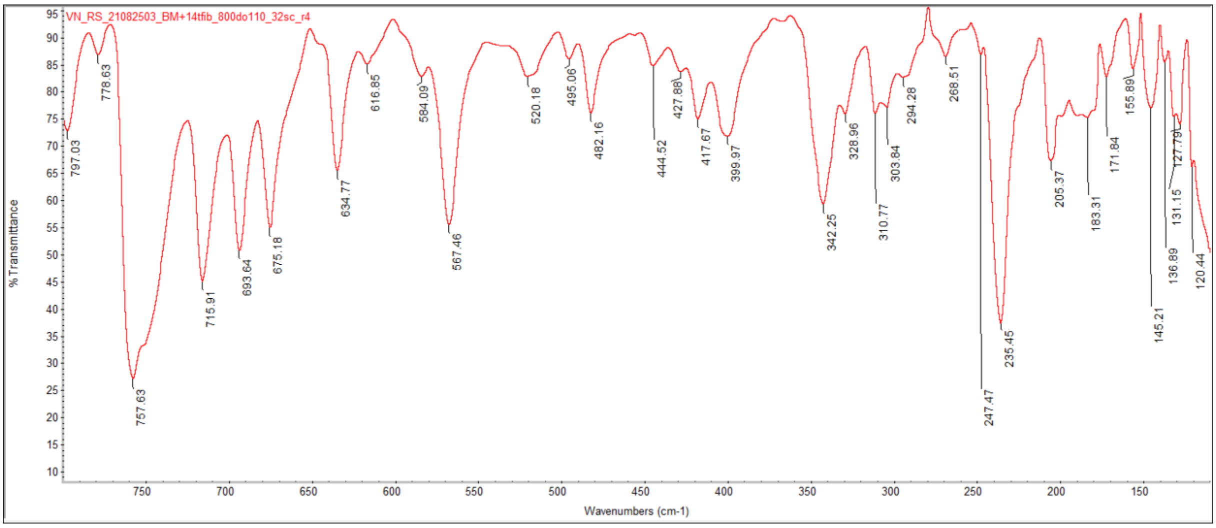

Figure S31. IR spectrum of (**BM**)$_2$(**14tfib**)$_5$ from 110 to 800 cm^{-1}

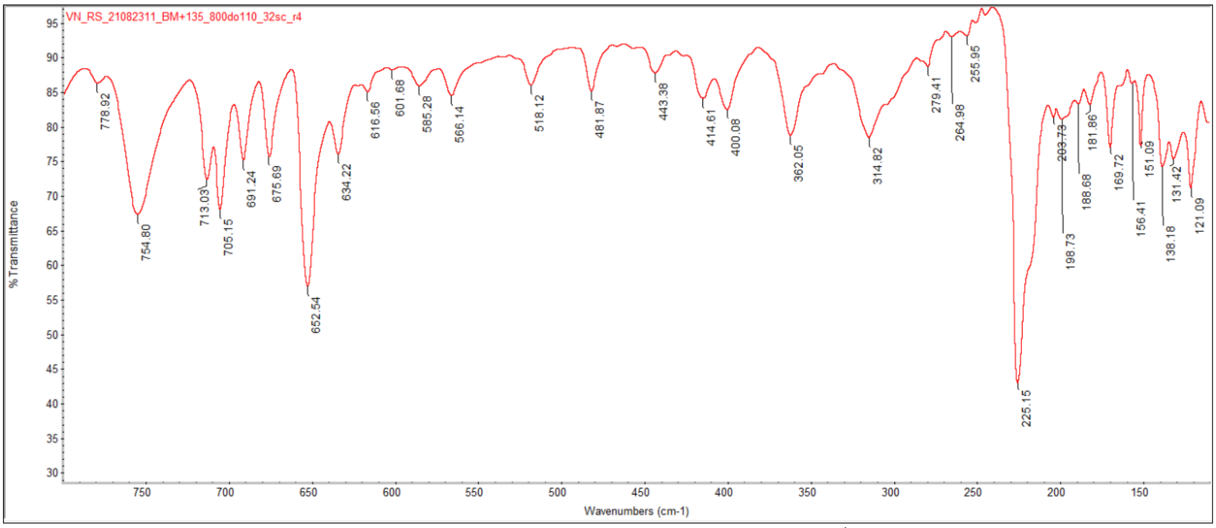

Figure S32. IR spectrum of (**BM**)(**135tfib**)$_2$ from 110 to 800 cm^{-1}

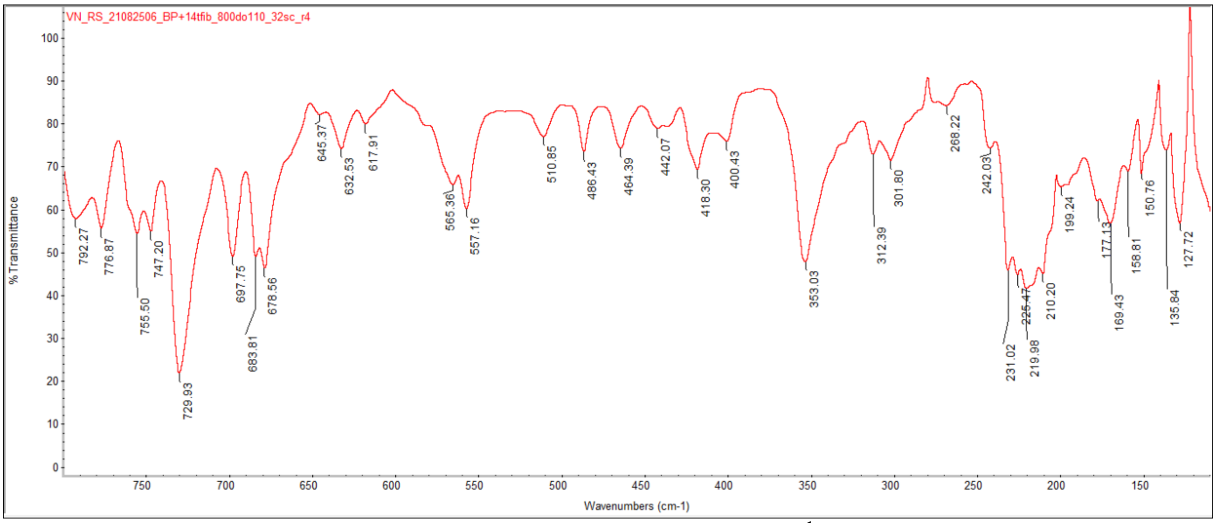

Figure S33. IR spectrum of (**BP**)(**14tfib**) from 110 to 800 cm^{-1}

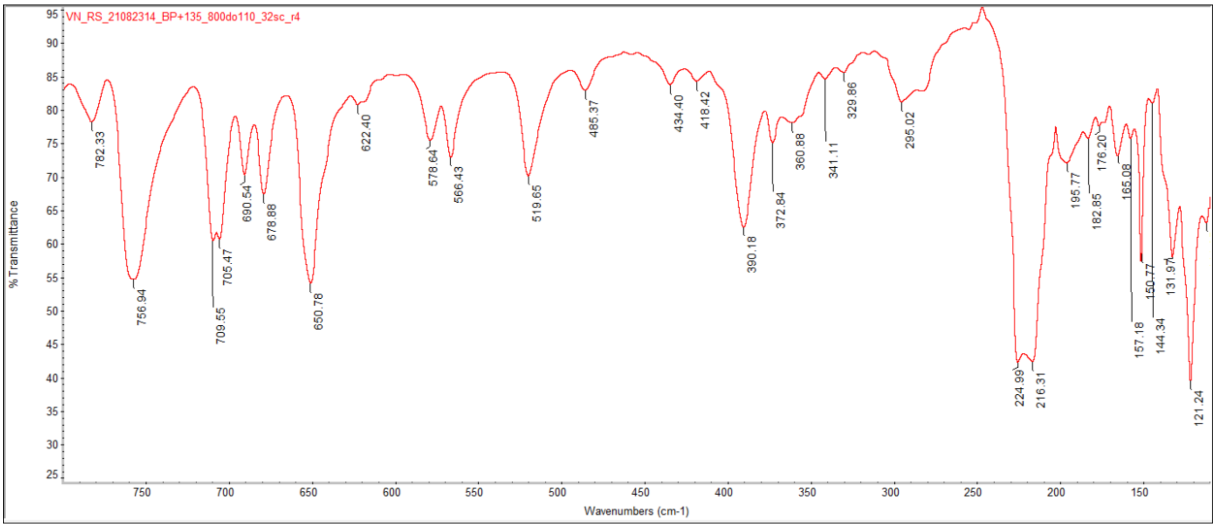

Figure S34. IR spectrum of (**BP**)(**135tfib**)$_2$ from 110 to 800 cm^{-1}

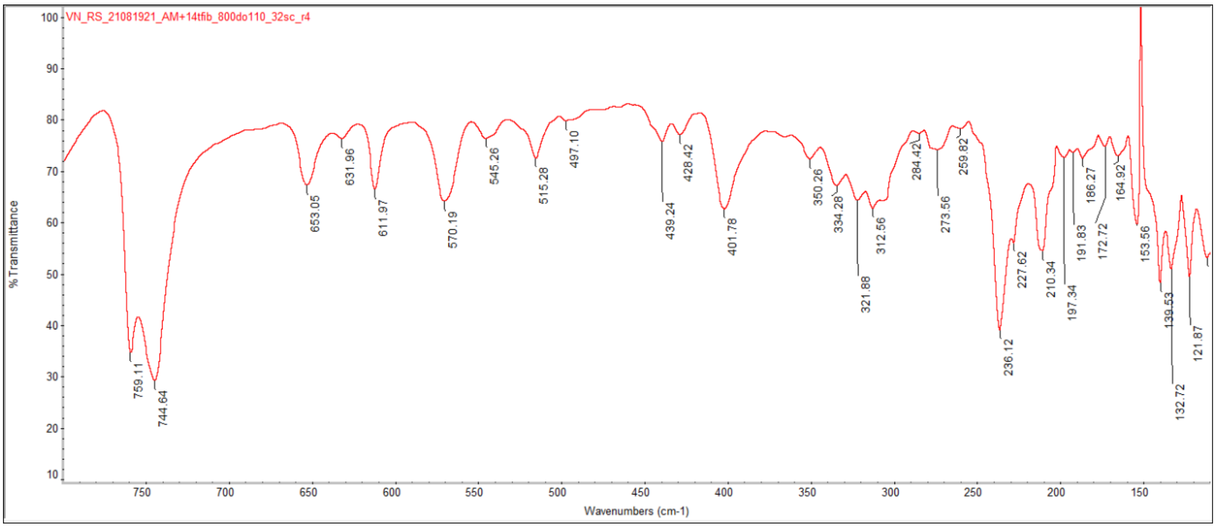

Figure S35. IR spectrum of (**AM**)(**14tfib**) from 110 to 800 cm^{-1}

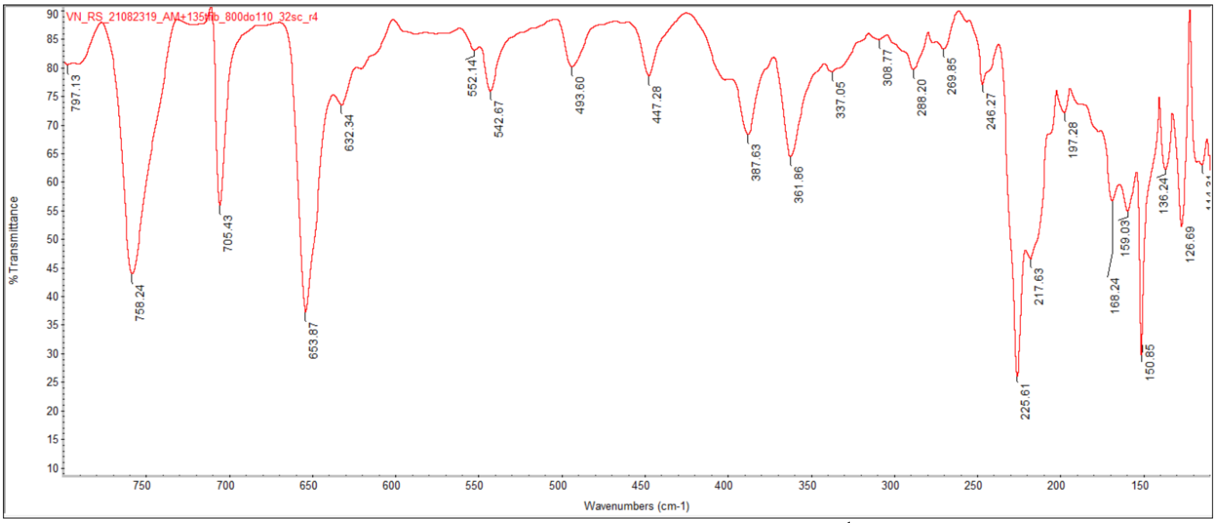

Figure S36. IR spectrum of (**AM**)(**135tfib**)$_2$ from 110 to 800 cm^{-1}

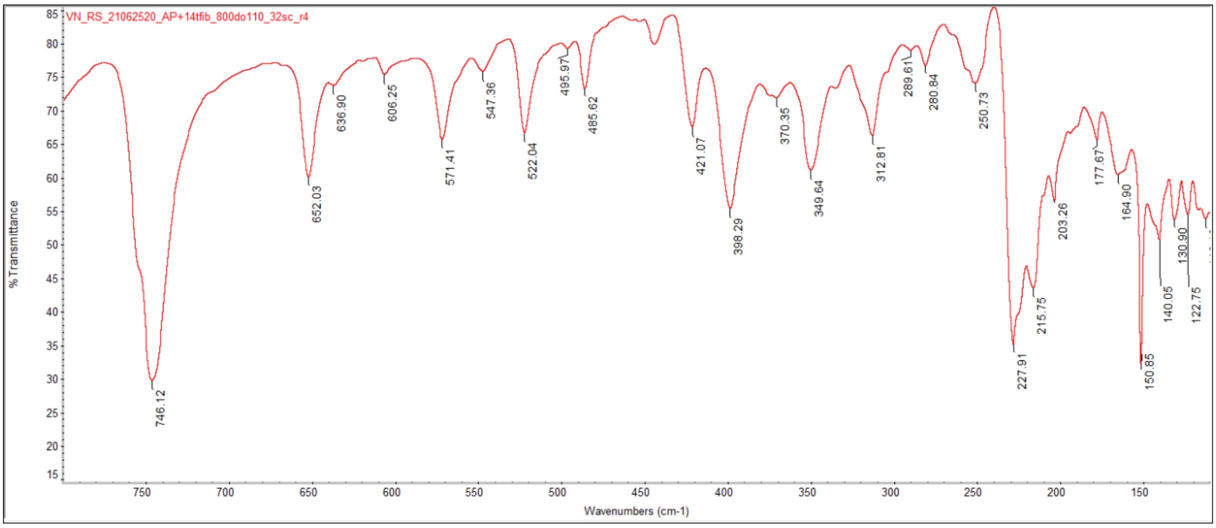

Figure S37. IR spectrum of (**AP**)(**14tfib**) from 110 to 800 cm^{-1}

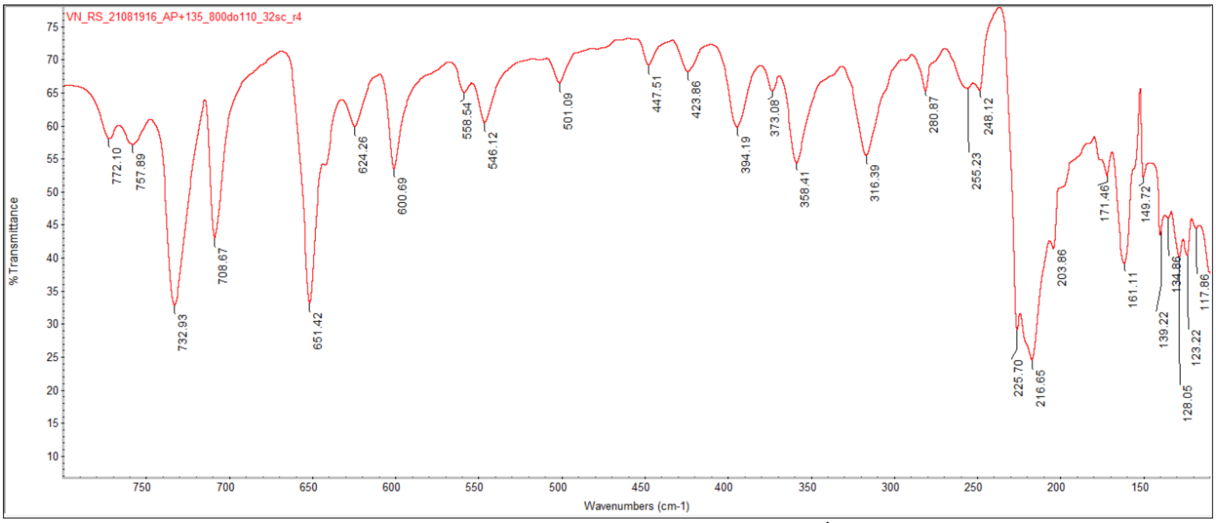

Figure S38. IR spectrum of (**AP**)(**135tfib**) from 110 to 800 cm^{-1}

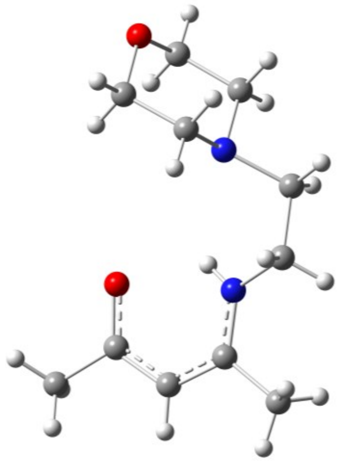

Figure S39. Optimized geometry of **AM** (bent).

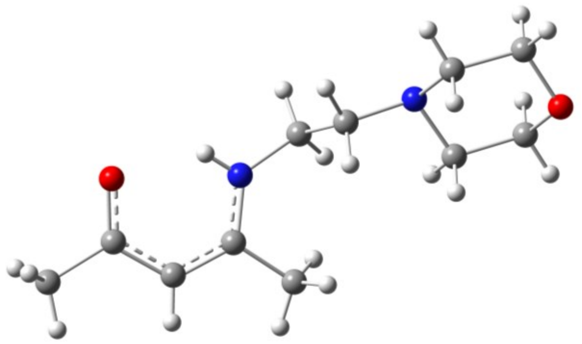

Figure S40. Optimized geometry of **AM** (extended conformation).

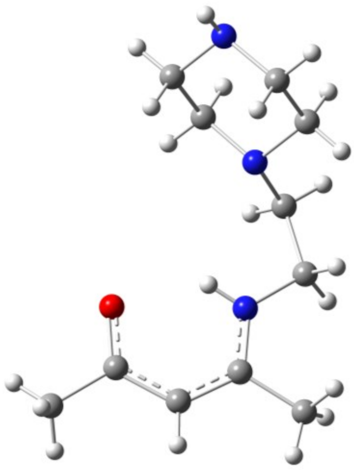

Figure S41. Optimized geometry of **AP**.

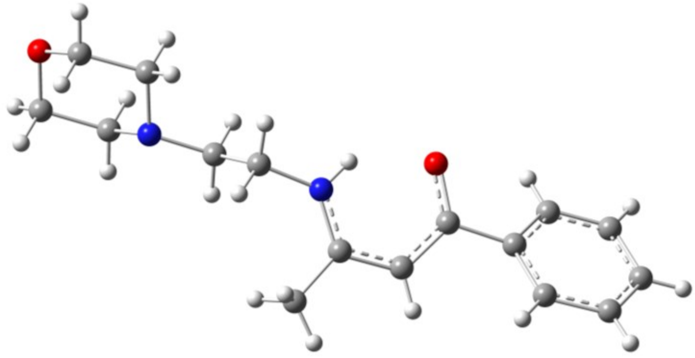

Figure S42. Optimized geometry of **BM**.

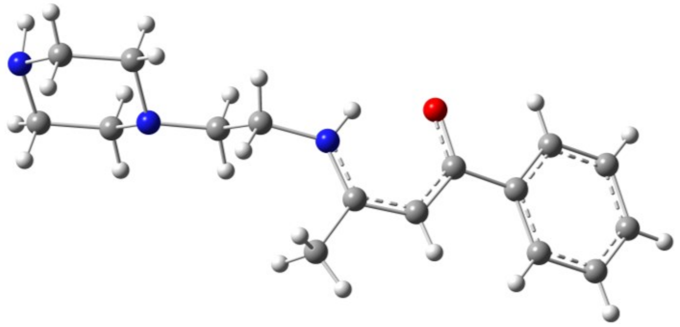

Figure S43. Optimized geometry of **BP**.
